# Orthorhombic Nb_2_O_5_ Decorated Carbon Nanoreactors Enable Bidirectionally Regulated Redox Behaviors in Room‐Temperature Na–S Batteries

**DOI:** 10.1002/advs.202206558

**Published:** 2022-12-05

**Authors:** Xiang Long Huang, Xiaofeng Zhang, Liujiang Zhou, Zaiping Guo, Hua Kun Liu, Shi Xue Dou, Zhiming Wang

**Affiliations:** ^1^ Institute of Fundamental and Frontier Sciences University of Electronic Science and Technology of China Chengdu 611731 China; ^2^ School of Physics University of Electronic Science and Technology of China Chengdu 611731 China; ^3^ School of Chemical Engineering & Advanced Materials The University of Adelaide Adelaide South Australia 5005 Australia; ^4^ Institute for Superconducting and Electronic Materials University of Wollongong New South Wales 2500 Australia; ^5^ Institute of Energy Materials Science University of Shanghai for Science and Technology Shanghai 200093 China

**Keywords:** bidirectional electrocatalyst, Na ion storage, Na–S batteries, orthorhombic Nb_2_O_5_, redox kinetics

## Abstract

Regulating redox kinetics is able to spur the great‐leap‐forward development of room‐temperature sodium–sulfur (RT Na–S) batteries, especially on propelling their Na‐ion storage capability. Here, an innovative metal oxide kinetics accelerator, orthorhombic Nb_2_O_5_ Na‐ion conductor, is proposed to functionalize porous carbon nanoreactors (CNR) for S cathodes. The Nb_2_O_5_ is shown to chemically immobilize sodium polysulfides via strong affinity. Theoretical and experimental evidence reveals that the Nb_2_O_5_ can bidirectionally regulate redox behaviors of S cathodes, which accelerates reduction conversions from polysulfides to sulfides as well as promotes oxidation reactions from sulfides to S. In situ and ex situ characterization techniques further verify its electrochemical lasting endurance in catalyzing S conversions. The well‐designed S cathode demonstrates a high specific capacity of 1377 mA h g^−1^ at 0.1 A g^−1^, outstanding rate capability of 405 mA h g^−1^ at 2 A g^−1^, and stable cyclability with a capacity retention of 617 mA h g^−1^ over 600 cycles at 0.5 A g^−1^. An ultralow capacity decay rate of 0.0193% per cycle is successfully realized, superior to those of current state‐of‐the‐art RT Na–S batteries. This design also suits emerging Na–Se batteries, which contribute to outstanding electrochemical performance as well.

## Graphical abstract

In this work, an intrinsic metal oxide electrocatalyst, orthorhombic Nb_2_O_5_, is revealed to bidirectionally regulate the redox behaviors of room‐temperature Na–S batteries, which are able to capture polysulfides via strong interactions, catalyze electrochemical reactions of S speciation, and improve redox kinetics. The Nb_2_O_5_‐decorated S cathode harvests excellent Na‐ion storage capability.

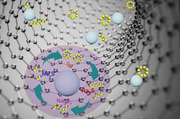

## 1 Introduction

Room‐temperature sodium–sulfur (RT Na–S) batteries potentially become one of large‐scale integrative electrochemical energy storage techniques due to some unique intrinsic advantages: abundant resources of Na and S, inexpensive price of key materials, high theoretical capacities of electrodes, and high energy density of devices.^[^ ^1^, ^2^, ^3^ ^]^ In nearly a decade of a rapid development road since 2010, a series of significant challenges are retarding the accomplishment of high‐performance RT Na–S batteries, mainly encompassing poor electronic conductivity of S and its solid‐phase discharge products, high solubility of polysulfide intermediates in liquid electrolytes, sluggish electrochemical kinetics of S species with Na ions, and dramatic volume variation of S cathodes during (de)sodiation process.^[^ ^4^, ^5^ ^]^ In recent years, delicate design of carbons with various fine nanostructures has significantly improved the reactivity of S species and alleviated the mechanical stress induced by volume expansion to a large extent.^[^ ^6^, ^7^ ^]^ Accordingly, it becomes the key to further developing Na–S batteries to both inhibit shuttle effect of sodium polysulfides (NaPSs) and boost thier electrochemical kinetics.

Metal oxides with rich surface sites have much promise to immobilize soluble NaPSs via polar–polar interactions so as to overcome the shuttle effect; whereas, most of the polar metal oxides exhibit too low intrinsic charge transfer capability to promote redox conversions of the surface‐bonded polysulfides, leading to generation of “dead S” and low capacities.^[^ ^8^, ^9^ ^]^ In order to improve the electron transfer ability of these polar metal oxides and catalyze phase transformations of surface‐bonded S species, some typical strategies have been put forward for enhanced RT Na–S chemistry. A typical approach is to elevate the charge transfer capability of metal oxides confined within carbon skeletons through tailoring their geometrical sizes, according to the well‐known nanosized effect.^[^ ^10^, ^11^ ^]^ Creating favoring defects via vacancies and/or elemental doping is also demonstrated to trigger the electrocatalytic effect of metal oxides toward S conversions through tuning their Fermi level.^[^ ^12^, ^13^ ^]^ Another useful method is to constitute heterostructures through deepening atomic‐scale interfacial coupling between metal oxides and substrate materials or epitaxially growing electrocatalysts on metal oxide surfaces.^[^ ^14^, ^15^ ^]^ Most often, these strategies necessitate cumbersome preparing and processing procedures and thus, require peculiar synthetic routes.

As a matter of fact, some metal oxides with decent charge transfer efficiency are recently verified to electrochemically catalyze polysulfide conversions or act as redox mediators, such as MoO_2_,^[^ ^16^ ^]^ MnO_2_,^[^ ^17^ ^]^ TiO,^[^ ^18^ ^]^ and Fe_2_O_3_.^[^ ^19^ ^]^ Their polar and catalytic properties are able to help not only capture soluble polysulfide intermediates via strong chemical bonding effect but also accelerate redox reactions of S species anchored on polar surfaces; thus, stabilizing S cathodes and propelling electrochemical performance. Moreover, for these catalytic metal oxides, less efforts on their structural design and chemical synthesis need to be made, leading to lower fabrication cost in contrast to those polar but nonconductive metal oxides that need extra structural/component modification for activating the electrocatalytic activity.

Among a great variety of metal oxide catalysts reported in metal–sulfur batteries, so far, orthorhombic Nb_2_O_5_ exhibits multiple unique merits for electrochemical energy storage, attributable to its open framework, layered structure, and chemical stability.^[^ ^20^ ^]^ Nb_2_O_5_ is an abundant material in nature and has a decent bulk electronic conductivity (3.4 × 10^−6^ S cm^−1^) compared with almost insulating metal oxides (e.g., TiO_2_).^[^ ^21^ ^]^ Nb–O crystalline structure leads to its strong sulfiophilicity for chemisorbing soluble polysulfides.^[^ ^22^ ^]^ Recent studies regarding Na‐ion batteries demonstrate that the Nb_2_O_5_ is a superior Na ion conductor, enabling ultrafast Na ion transport not only at their surfaces but also in the bulk.^[^ ^23^, ^24^ ^]^ In addition, orthorhombic Nb_2_O_5_ is expected to serve as an electrochemically stable redox promotor in a long‐term cycling process due to its high corrosion resistance.^[^ ^22^ ^]^ To our best knowledge, such an ideal redox accelerator has still not been investigated in RT Na–S batteries, hitherto. The underlying viability of orthorhombic Nb_2_O_5_ in RT Na–S batteries can be inferred, given its high performance in similar lithium–sulfur (Li–S) batteries.

**Figure 1:**
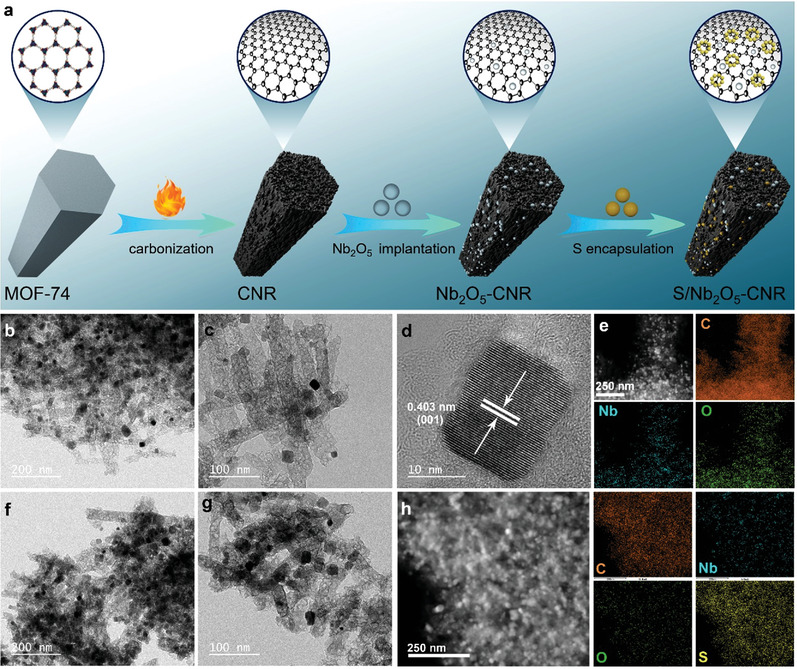
Synthesis and morphologies of samples. a) Schematic illustration of the synthesis of the S/Nb_2_O_5_–CNR. b–e) TEM, HRTEM, STEM images, and corresponding EDS elemental mappings of the Nb_2_O_5_–CNR. f,g) TEM images of the S/Nb_2_O_5_–CNR composite. h) STEM image and EDS elemental mappings of the S/Nb_2_O_5_–CNR composite.

In this work, we elaborate a rod‐like carbon nanoreactor (CNR) as the substrate material for both Nb_2_O_5_ and S. **Figure** **1**a sufficiently embodies the design concept and synthetic process of the elaborated S electrode material. The CNR featuring a hierarchically porous structure functions as a container for S guest, a buffer of volume change, and more significantly, as a carrier platform of electrochemical reactions. In previous works on RT Na–S batteries, metal oxides such as TiO_2_ could not catalyze redox conversions of S due to poor electronic conductivity. Differently, the orthorhombic Nb_2_O_5_ with a superior Na ion conductivity is evidenced as both a polar NaPS adsorbent and an excellent redox regulator, enabling to both inhibit polysulfide shuttling and accelerate conversion kinetics in working RT Na–S batteries. A series of electrocatalysts such as metal sulfides/carbides is shown to facilitate reduction reactions of S/polysulfides; however, catalytic oxidation reactions from Na_2_S/polysulfides to S are still less reported and proved, to the best of our knowledge. Impressively, theoretical and experimental analysis indicates that the orthorhombic Nb_2_O_5_ can bidirectionally regulate redox behaviors of S cathodes, that is, both catalyzing reduction reactions from polysulfides to sulfides and promoting oxidation reactions from sodium sulfides to S. Moreover, uniformly dispersed orthorhombic Nb_2_O_5_ renders the whole CNR an ultrafast Na ion conduction network, in favor of solid–solid conversions among insoluble solid‐phase products via fast transport of Na ions. Electrochemical endurance of the orthorhombic Nb_2_O_5_ is disclosed via in‐situ XRD and ex‐situ XPS results, which contributes to lasting catalytic conversions of S cathodes with improved kinetics. As a result of the functional synergy of micro/nanostructures and chemical compositions, the S/Nb_2_O_5_–CNR cathode harvests a high discharge specific capacity of 1377 mA h g^−1^ at 0.1 A g^−1^, excellent rate capability of 405 mA h g^−1^ at 2 A g^−1^, and outstanding cyclability with a capacity retention of 617 mA h g^−1^ after 600 cycles at 0.5 A g^−1^. In addition, it may be generalized to other similar rechargeable metal–chalcogen batteries, exemplifying Na–Se batteries in this work. This work reveals the stable kinetics promotion effect of orthorhombic Nb_2_O_5_ for S conversion chemistry and highlights the importance of functionalized design of cathode materials for achieving high‐performance RT Na–S batteries.

## 2 Results and Discussion

### 2.1 Materials Design and Characterization

A lot of research literature demonstrates that hierarchical nanopores hold outstanding confinement ability and encapsulation effect for S guest.^[^ ^25^, ^26^, ^27^ ^]^ In order to achieve uniform dispersion of S molecules and ideal immobilization toward them, an easily synthesized Zn‐based metal–organic framework (MOF‐74) was directly harnessed as a template to prepare a hierarchically porous carbon (Figure 1a). XRD pattern in Figure S1, Supporting Information; SEM image in Figure S2, Supporting Information; and TEM images in Figure S3, Supporting Information; jointly demonstrate that the MOF‐74 is in good accordance with previous reports,^[^ ^28^, ^29^ ^]^ confirming its successful synthesis. In the subsequent carbonization process, Zn ions in the precursor were reduced into gas‐state metallic Zn and taken away by the Argon flow, while the rest of the organic components were pyrolyzed into porous carbon. Eventually, the precursor was successfully converted to structure‐stable hierarchically porous carbon that functions as a carbon nanoreactor (CNR). The CNR integrally inherits the nanorod‐like microscopic morphology of the precursor and more importantly, possesses abundant hollow nanovoids, as evidenced by Figure S4, Supporting Information. The nanorod‐like architecture provides electrically conductive pathways for rapid electron transport, favoring fast kinetics in multielectron‐involved electrochemical reactions.

Next, Nb_2_O_5_ nanoparticles were implanted onto the whole CNR skeleton to endow it with multifunctional feature. As shown in Figure 1b, the resultant ultrafine Nb_2_O_5_ nanoparticles are uniformly dispersed onto the CNR substrate, which is favorable for multiple‐site adsorption and interface conversions of intermediates. Despite the incorporation of plentiful Nb_2_O_5_ nanoparticles, the CNR still retains its original morphologies of porous nanorods without any structural change (Figure 1c), suggesting its structural stability. Furthermore, an interplanar distance of 0.403 nm can be clearly observed from Figure 1d, which exactly corresponds to the (001) plane of orthorhombic Nb_2_O_5_. The scanning transmission electron microscopy (STEM) accompanied with the corresponding energy dispersive spectroscopy (EDS) was utilized to detect the elemental distributions of the Nb_2_O_5_–CNR composite (Figure 1e). It proves the homogeneous dispersion of Nb_2_O_5_ into the CNR, evidently indicating effective implantation of Nb_2_O_5_ into carbon matrix.

X‐ray diffraction (XRD) was operated so as to analyze the phase components of the as‐prepared materials (Figure 1a,b). There appear two obvious characteristic peaks located 26° and 44° in the pattern of CNR, where the former is related to graphitic carbon diffraction (002) while the latter corresponds to (101) facet of carbons.^[^ ^30^ ^]^ It suggests the partially graphitized amorphous carbon nature of the CNR. Any peaks associated to metallic Zn are not observed, indicating complete removal of Zn and high purity of the CNR. The XRD pattern of CNR shows that the highly crystalline precursor MOF‐74 is fully converted to amorphous carbon through a facile high‐temperature carbonization treatment. The implantation of Nb_2_O_5_ nanoparticles leads to generation of many new characteristic peaks with high intensity that are very coincident with those of PDF# 30–0873, which fully identifies its orthorhombic phase and high crystallinity. The thermogravimetric analysis (TGA) result (Figure S5, Supporting Information) indicates that the content of orthorhombic Nb_2_O_5_ is 16.8% in the Nb_2_O_5_–CNR. As sublimed S is composited with the Nb_2_O_5_–CNR, the peak intensity of orthorhombic Nb_2_O_5_ is slightly reduced compared with the Nb_2_O_5_–CNR substrate. At the same time, there is lack of obvious characteristic peaks ascribed to S in the XRD pattern of the S/Nb_2_O_5_–CNR composite. XRD pattern of pristine sublimed S is displayed in Figure S6, Supporting Information. Such a case discloses that S is evenly infused into hollow nanovoids of the CNR. Good encapsulation of S into carbon nanopores is beneficial to suppressing dissolution and shuttling of polysulfide intermediates.

**Figure 2:**
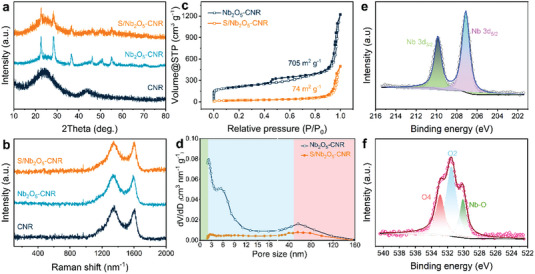
Structures, compositions, and porosity of materials. a,b) XRD patterns and Raman spectra of CNR, Nb_2_O_5_–CNR, and S/Nb_2_O_5_–CNR composite. c,d) Nitrogen adsorption–desorption isotherms and pore size distributions of the Nb_2_O_5_–CNR and S/Nb_2_O_5_–CNR composites. e,f) XPS spectra of Nb 3d and O 1s.

Raman spectroscopy with an examined wavelength range from 100 to 2000 nm was implemented to reveal micro/nanostructure nature of the CNR, Nb_2_O_5_–CNR, and S/Nb_2_O_5_–CNR (**Figure** **2**b). Two clear peaks positioning at 1350 and 1599 cm^−1^ can be witnessed from the Raman spectra of all samples, which actually represent D band and G band of carbons, respectively.^[^ ^31^ ^]^ The D band acts as an indicator of disorder structure of amorphous carbon while the G band suggests the graphitization structure of carbon. The intensity ratio of D band and G band (*I*_D_/*I*_G_) can usually quantificationally reflect the graphitization level of carbon and thus, unveils its defective nature.^[^ ^32^, ^33^ ^]^ The calculated *I*_D_/*I*_G_ value of CNR is about 1.08, fully demonstrating its partially graphitized defective carbon structure and further validating the aforementioned XRD analysis result. Despite post‐treatments via impregnation of both Nb_2_O_5_ and S, the *I*_D_/*I*_G_ value of sample materials remains unchanged, consequently proving structural/chemical stability of the CNR. Notably, no characteristic peaks from pure S (Figure S7, Supporting Information) are detected in the Raman spectra of the S/Nb_2_O_5_–CNR, indicating successful and sufficient infiltration of S into porous carbon once again. The S content in the S/Nb_2_O_5_–CNR composite is 42% (Figure 2c), as determined by the TGA result.

Considering the decisive impact of the porous structure on electrochemical performance of S cathodes, we utilized TEM and Brunauer–Emmert–Teller (BET) method to investigate porous nature of the Nb_2_O_5_–CNR and S/Nb_2_O_5_–CNR. There are a lot of observable nanopores with different sizes in TEM images of the Nb_2_O_5_–CNR (Figure S8, Supporting Information). The extensive distribution of these nanopores into the CNR conduces to improving the electrochemical reactivity of S molecules confined within them. Figure 2c simultaneously depicts nitrogen adsorption–desorption isotherms of the Nb_2_O_5_–CNR and S/Nb_2_O_5_–CNR composites. For the Nb_2_O_5_–CNR, a dramatic increase in N_2_ adsorption at low pressure region indicates the presence of micropores while a clear hysteresis at high pressure region is a signal of mesopores.^[^ ^34^ ^]^ It exhibits a high specific surface area of 705 m^2^ g^−1^. For the S/Nb_2_O_5_–CNR with a far smaller specific surface area of 74 m^2^ g^−1^, the N_2_ adsorption feature at low pressure region obviously disappears and the hysteresis loop at high pressure region is also significantly reduced. This suggests that a great many nanopores are padded with the active materials. The corresponding pore‐size distribution in Figure 2d further reveals the porosity of all materials. In the distribution profile of the Nb_2_O_5_–CNR, micropores, mesopores, and macropores can be clearly witnessed. The sizes of these nanopores center at 1.5, 6, and 60 nm, respectively. These nanopores rapidly disappear along with the S impregnation, but some nanopores remain. These residual nanopores can offer more reactive sites for electrochemical reactions and limit the shuttling of intermediate products. Table S1, Supporting Information, summarizes the pore volume and specific surface areas of all the samples.

X‐ray photoelectron spectroscopy (XPS) was further employed to detect the compositions of the S/Nb_2_O_5_–CNR and its surface chemical structures. A series of characteristic peaks in the wide‐survey XPS spectrum are shown in Figure S9, Supporting Information, which correspond to O, C, S, and Nb element, respectively. Nb and O elements are further fitted to multiple deconvolutions for determining their chemical valence states. Two strong peaks located at 207.1 and 209.9 eV fully indicate its valence of Nb^5+^ in XPS spectra of Nb 3d (Figure 2e).^[^ ^35^ ^]^ In O 1s spectra (Figure 2f), there are three evident sub peaks, which originate from Nb—O bonds, hydroxylated groups, and adsorbed moisture on carbon surface, respectively.^[^ ^36^ ^]^

Generally speaking, the above characterization results verify the successful fabrication of the elaborate Nb_2_O_5_ nanoparticle‐decorated carbon nanorods, which are expected to be multifunctional nanoreactors for RT Na—S batteries due to their advantages in chemical compositions and micro/nanostructures. First, the unique hierarchically porous nature is able to afford rich nanovoids for S storage and circumvent the dramatic volume change of S species during cycling. Second, nanorods with a high specific surface area can facilitate the rapid electron transfer along with its natural pathways and accessible Na ion adsorption. Third, polar Na‐ion conductor Nb_2_O_5_ functionalizes carbon nanorods with excellent sodiophilicity and sulfiphilicity to trap the S species as well as promote the electrochemical kinetics via regulating redox reactions in working RT Na‐S batteries.

### 2.2 Chemical Affinity and Catalytic Nature of Orthorhombic Nb_2_O_5_

**Figure 3:**
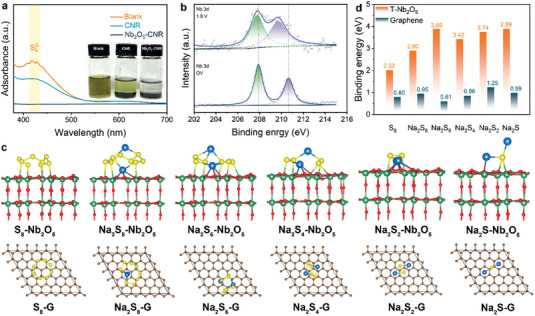
Interactions between Nb_2_O_5_ and polysulfides. a) UV–visible adsorption spectra of the Na_2_S_6_ solution before and after soaking with different materials (inset: photographs for different Na_2_S_6_ solutions). b) XPS spectra of Nb 3d before and after the S/Nb_2_O_5_–CNR is discharged. c) Optimized adsorption configurations of various Na_2_S*_x_* on orthorhombic Nb_2_O_5_ and graphene (G). d) Comparison of binding energies between various Na_2_S*_x_* and different substrates (Nb_2_O_5_ and graphene G).

Considering the significance of immobilizing/catalyzing polysulfides for stabilizing S cathodes, the adsorbent role of Nb_2_O_5_ for NaPSs is jointly verified via theoretical and experimental evidence. For visual observation of adsorption ability of substrates toward polysulfides, a Na_2_S_6_ solution as a representative of polysulfides is prepared to load the identical mass of target materials. The color change of Na_2_S_6_ solutions is typically deemed as a monitor of adsorption ability of adsorbents. As displayed in the inset of **Figure** **3**a, the pristine Na_2_S_6_ solution exhibits a transparent yellow color, the Na_2_S_6_ solution with the CNR demonstrates a slight decoloration, and the Na_2_S_6_ solution with the Nb_2_O_5_—CNR becomes colorless. The slight decoloration phenomenon shows the greatly limited adsorption ability of the CNR. In contrast to that, the Nb_2_O_5_—CNR shows a much stronger adsorption ability, which results from the chemical affinity of Nb_2_O_5_ toward polysulfides. This result is supported by UV–vis spectra in Figure 3a, which can usually show the Na_2_S_6_ residual via the polysulfide moiety absorbance intensity and thus, explain the adsorption ability of additives. The broad band at 424 nm ascribed to Na_2_S_6_ from the pristine Na_2_S_6_ solution completely vanishes after adding the Nb_2_O_5_–CNR, validating the strong adsorption capability of Nb_2_O_5_ to polysulfides.

For revealing the adsorption effect of Nb_2_O_5_ toward polysulfides, XPS spectra were carried out on the S electrode before (open voltage, OV) and after discharging to 1.8 V so as to analyze the chemical bonding between polysulfides and Nb_2_O_5_. It can be clearly found from Figure 3b that after discharging, two characteristic peaks ascribed to Nb 3d evidently shift to lower binding energy. The peak position shift is the result of the electron transfer between Nb_2_O_5_ and polysulfides in working S cathode to form Nb—S bonds, according to the previous reports.^[^ ^21^ ^]^ In XPS spectra of O 1s (Figure S11, Supporting Information), a peak attributed to Na—O bond is shown after discharging the S cathode to 1.8 V.^[^ ^37^ ^]^ Therefore, it can be concluded from these results that the Nb_2_O_5_ enables chemically bonding Na_2_S*_x_* via the mechanism of Nb—S and Na—O bonds, thereby confirming the sodiophilic (Na—O bonds) and sulfiphilic (Nb—S bonds) nature of Nb_2_O_5_. XPS spectra were conducted on the S cathode after discharging to cut‐off voltage (0.8 V). Figure S12, Supporting Information, shows the XPS spectra of Nb 3d. The two dominant peaks returned to the original positions at the OV state. This means both easy adsorption and desorption of Na_2_S*_x_* on the Nb_2_O_5_ surface so that no (or less) dead “S” was formed and accumulated, rendering high electrochemical reversibility of Na_2_S*_x_*.

Density‐functional theory (DFT) calculations were performed, aiming to disclose the binding energies between Na_2_S*_x_* and substrate materials and further verifying the strong chemical affinity of Nb_2_O_5_ for polysulfides. Figure 3c and Figure S13 (Supporting Information) display the adsorption configurations between various polysulfides and carbon/Nb_2_O_5_, separately. The interactions between pure carbon and Na_2_S*_x_* rely on weak van der Waals force (physical adsorption). The binding energies of S_8_, Na_2_S_8_, Na_2_S_6_, Na_2_S_4_, Na_2_S_2_, and Na_2_S on pure carbon are 0.80, 0.95, 0.61, 0.86, 1.25, and 0.99 eV (Figure 3d), respectively. These low binding energies indicate the stable and inactive surface properties of pure carbon substrate for S species. Differently, the orthorhombic Nb_2_O_5_ surface interacts with various S species via strong chemical adsorption force, which exhibits greatly enhanced binding energies of 2.02, 2.90, 3.89, 3.42, 3.74, and 3.89 eV, respectively. Notably, the binding energy of Nb_2_O_5_ for intermediate product Na_2_S_4_ reaches up to 3.89 eV, which contributes to stable catalytic conversions from Na_2_S_4_ to insoluble products (Na_2_S_2_/Na_2_S).

**Figure 4:**
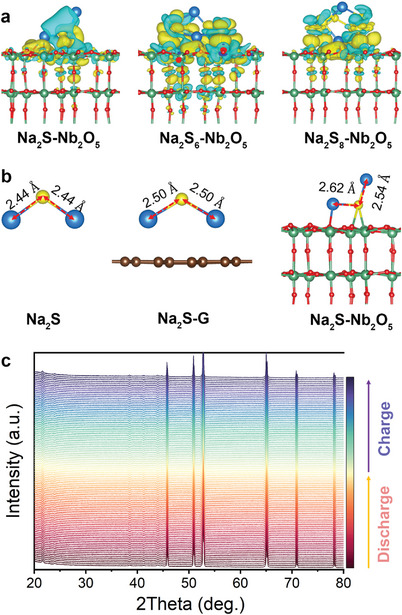
Electrocatalytic nature of orthorhombic Nb_2_O_5_ for RT Na–S chemistry. a) Charge density differences for Na_2_S, Na_2_S_6_, and Na_2_S_8_ molecules adsorbed on the Nb_2_O_5_. The iso‐surfaces are all 0.001 e bohr^−3^. b) Bond length variation of the Na_2_S molecule at different states. The yellow and blue represent electron accumulation and depletion, respectively. c) In‐situ XRD pattern of the S/Nb_2_O_5_–CNR during discharge–charge process.

Catalytic conversions of S electrochemistry are highly dependent on electron transport properties, as revealed in previous reports.^[^ ^38^, ^39^, ^40^ ^]^ We first employed DFT calculations to disclose charge transfer feature of the Na_2_S*_x_*–Nb_2_O_5_ adsorption system. The charge density distribution between pure carbon and Na_2_S/Na_2_S_6_/Na_2_S_8_ is obviously interrupted without charge accumulation and with formation of a wide gap, demonstrating that the pure carbon is unable to offer an electron‐conducting pathway to subsequent reduction of the bonded polysulfides (Figure S14, Supporting Information). In comparison, the charge density distribution between Nb_2_O_5_ and Na_2_S/Na_2_S_6_/Na_2_S_8_ is continuous, which can establish a smooth conductive channel for Na_2_S*_x_* conversions. Besides, a large number of charges tending to gather at the Nb—S and Na—O bonds can enhance the chemical adsorption of S species as well as promote the multisite catalytic conversions from Na_2_S*_x_* to Na_2_S (**Figure** **4**a).This is certified by the obvious cathodic peak shift in CV curves in Figure S15 (Supporting Information), where reduction peaks of the S/Nb_2_O_5_—CNR shift to higher potential compared with that of the S/CNR, suggesting kinetically faster conversions of Na_2_S*_x_* with the catalysis of Nb_2_O_5_. Meanwhile, the discharge–charge profiles of the S/Nb_2_O_5_–CNR show a smaller voltage polarization than that of the S/CNR (Figure S16, Supporting Information), further proving the Nb_2_O_5_‐induced catalytic effect for polysulfide conversions.

The Na—S bond length of Na_2_S at different states is analyzed to investigate their decomposition during desodiation process (Figure 4b). The Na—S bond length in the pristine Na_2_S is 2.44 Å. After depositing on the pure carbon surface, the bond length slightly increases to 2.50 Å, while after adsorbing on the Nb_2_O_5_ surface, it is significantly lengthened to 2.62 Å. The lengthened Na—S bond suggests the weakened interactions between Na_2_S and substrates and easier decomposition. Therefore, the incorporation of Nb_2_O_5_ can facilitate the oxidation reactions from Na_2_S to S_8_.

The electrochemical durability of catalysts is a crucial factor for stabilizing catalytic S electrodes. To the best of our knowledge, more research interest is focused on designing novel electrocatalysts and validating their catalytic activity for S conversions but less attention is paid to unveiling their endurance during the electrochemical process, hitherto. Most often, electrocatalysts for S chemistry can be divided into three types: 1) phase‐stable catalysts that always preserve their phase compositions in the entire discharge–charge process;^[^ ^41^, ^42^, ^43^ ^]^ 2) pre‐catalysts that are converted to other catalytic phases via defect filling and/or polysulfide corrosion;^[^ ^44^, ^45^, ^46^ ^]^ 3) catalysts that will be partially sodiated during discharge–charge process to generate stronger catalytic activity.^[^ ^47^, ^48^ ^]^ Herein, electrochemical stability of the orthorhombic Nb_2_O_5_ catalyst during discharge–charge process is examined via in‐situ XRD technique (Figure 4c) After a consecutive discharging–charging test, all the characteristic peaks of orthorhombic Nb_2_O_5_ remain unchanged in the S cathode, with lasting peak intensity and without any shift. This is highly consistent with the afore XPS analysis results. In‐situ XRD patterns and ex‐situ XPS spectra fully indicate the high electrochemical corrosion resistance of orthorhombic Nb_2_O_5_ in catalyzing RT Na—S chemistry and demonstrating its durable electrocatalytic function.

As a whole, substantial experimental and theoretical evidence proves the strong chemical affinity of orthorhombic Nb_2_O_5_ for a series of S species ranging from S_8_ to Na_2_S*_x_* and to Na_2_S. Impressively, orthorhombic Nb_2_O_5_ is revealed as an electrochemically stable bidirectional electrocatalyst for S conversions, which can not only accelerate the reduction conversions from chain‐like polysulfides to Na_2_S but also promote oxidation conversions from Na_2_S to S_8_. It strong adsorptive and catalytic ability can inhibit the shuttle effect and reinforce redox kinetics.

### 2.3 Na‐Ion Storage Properties of the S/Nb_2_O_5_–CNR Composite

**Figure 5:**
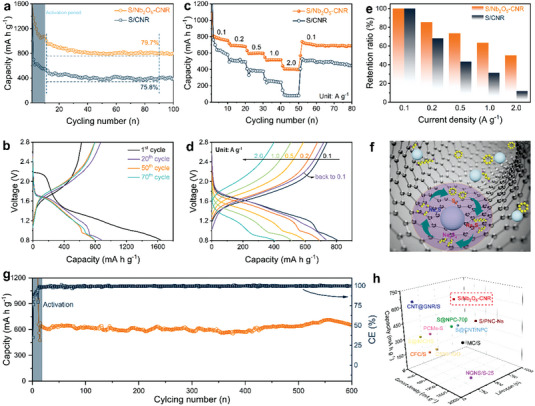
Electrochemical performance of RT Na–S batteries. a) Cycling performance at 0.1 A g^−1^. b) Discharge–charge profiles at 0.1 A g^−1^. c) Rate capabilities of the S/Nb_2_O_5_–CNR and S/CNR at various current densities. d) Discharge–charge profiles at various current densities. e) A comparison of capacity retention ratio along with increased rates. f) Schematic illustration of promoted redox conversion. g) Long‐term cycling performance at 0.5 A g^−1^. h) Comparison of prolonged cycling performance for S/carbon cathodes.

Based on the synergistic design of structures and compositions for the S/Nb_2_O_5_–CNR composite, it is expected to serve as an ideal electrode material for high‐performance Na‐ion storage. Its cycling capacities are first evaluated by galvanostatic discharge/charge tests at a small current density of 0.1 A g^−1^ (**Figure** **5**a). The S/Nb_2_O_5_–CNR composite realizes a high reversible discharge specific capacity of 1377 mA h g^−1^, which is stabilized with a capacity retention of 800 mA h g^−1^ after 100 cycles. Nevertheless, for the S/CNR composite without the Nb_2_O_5_ incorporation, it demonstrates much worse cycling performance at the same rate, with a capacity retention of 389 mA h g^−1^ after 100 cycles. Besides, the Coulombic efficiency (CE) of the former is higher than that of the latter (Figure S15, Supporting Information). The discharge–charge profiles in Figure 5b are highly overlapped with almost stable capacity, indicating the high reversibility of electrochemical reactions. We can witness that the capacity contribution of the involved S cathodes is mainly from reversible electrochemical conversions of active S because the substrate materials (Nb_2_O_5_–CNR and CNR) show negligible capacities below 50 mA h g^−1^ (Figures S16 and S17, Supporting Information). Taken together, the improved cycling capacity and CE of the S/Nb_2_O_5_–CNR composite confirms the key role of Nb_2_O_5_ in elevating S utilization and promoting redox conversions of S. It is notable that rapid capacity fading and very low CE occurs at the first few cycles in both the S/Nb_2_O_5_–CNR and S/CNR composite, which arises from construction of solid–electrolyte‐interface (SEI)/cathode–electrolyte interface (CEI) film via polysulfide‐associated parasitic reactions, well recognized in the previous literature about RT Na–S batteries.^[^ ^41^, ^43^, ^49^ ^]^ The stable CEI film can function as a protective layer for S cathodes to help inhibit the loss of the active materials to some extent, achieving more stable capacity and higher CE at the following cycles.^[^ ^50^, ^51^ ^]^ Thus, a technique conflict is that the construction of CEI layers can derive a protective layer for long‐term stable cycling but leads to a low capacity and CE during the initial period. In future research, a good solution (e.g., electrolyte regulation, electrode coating layers, and material structure design etc.) needs to be proposed in order to construct stable CEI film without S/electrolyte consumption but with improved cycling capacity and CE.

Outstanding rate capabilities are also obtained with the well‐designed S/Nb_2_O_5_–CNR composite (Figure 5c,d). The S/Nb_2_O_5_–CNR composite delivers reversible capacities of 809, 693, 595, 514, and 405 mA h g^−1^ at current densities ranging from 0.1 to 2.0 A g^−1^, while in contrast, the S–CNR composite delivers much lower capacities of 716, 520, 330, 239, and 89 mA h g^−1^ at the corresponding rates. Upon returning to the small current density of 0.1 A g^−1^, the S/Nb_2_O_5_–CNR composite obtains a far higher reversible capacity of 740 mA h g^−1^ than that of S–CNR composite (513 mA h g^−1^). The corresponding CE is displayed in Figure S18 (Supporting Information). Furthermore, the discharge–charge profiles obtained at various current densities present similar features and small polarization accompanied with fast reaction kinetics. In comparison, the S–CNR composite shows polarization‐large discharge–charge profiles (Figure S19, Supporting Information), especially at large current densities, indicating its poor kinetics nature. The decreased polarization can be attributed to faster interfacial charge transport due to the incorporation of Nb_2_O_5_ regulator, as evidenced by EIS spectra before and after cycling (Figure S20, Supporting Information). In addition, Na‐ion diffusion after cycling is obviously reinforced due to the higher sloping in low‐frequency region, which should arise from the superior Na‐ion conducting nature of Nb_2_O_5_. In order to profoundly understand the rate capability, the capacity retention ratios along with increased current densities in comparison to the baseline at 0.1 A g^−1^ are introduced (Figure 5f). In terms of the S/Nb_2_O_5_–CNR composite, the capacity retention ratios of 85.6%, 73.5%, 63.5%, and 50.1% are acquired at 0.2, 0.5, 1, and 2 A g^−1^, respectively. In terms of the S–CNR composite, the capacity retention ratios of 68.4%, 43.4%, 31.5%, and 11.7% are acquired at 0.2, 0.5, 1, and 2 A g^−1^, respectively.

Apart from the evaluation for basic Na‐ion storage performance, an appealing S cathode is required to possess a long‐term cyclability for its practical applications. We expect that the elaborate S/Nb_2_O_5_–CNR cathode can exhibit a desirable cycling stability for RT Na–S batteries so that a decent current density of 0.5 A g^−1^ is selected to perform the discharge–charge tests (Figure 5g). After a short activation process at 0.1 A g^−1^ enabling stable CEI protective layer, the S cathode exhibits a reversible specific capacity of 696 mA h g^−1^ and maintains a specific capacity of 617 mA h g^−1^ after 600 cycles at 0.5 A g^−1^ with an ultralow attenuation rate of 0.0193% per cycle. Besides, a continuously stable CE close to 100% is realized during prolonged cycling process. In order to testify the superiority and advancement of cyclability obtained via the S/Nb_2_O_5_–CNR, it is carefully compared with the state‐of‐art RT Na–S batteries with S/carbon cathodes (Figure 5h) and catalyst‐decorated S/carbon cathodes (Figure S21, Supporting Information). The comparison results clearly demonstrate that its performance is superior to most of the literature regarding current RT Na–S batteries.

Postmortem analysis experiments were conducted for separators and S electrodes that were disassembled from cycled coin cells to disclose why to acquire excellent sodium‐storage properties. In general, the separator color after cycling is considered as a direct describer of S electrode stability because their color can reflect the dissolution and shuttling of polysulfides in working RT Na–S batteries. The separator from the cell with the S/Nb_2_O_5_–CNR presents a white color in contrast to the obvious yellow color of that with the S/CNR (Figure S22, Supporting Information). This suggests the well‐inhibited polysulfide shuttling phenomenon with the help of Nb_2_O_5_. Furthermore, we took an observation of microscopic structures of materials after cycling via TEM and HRTEM. The results in Figure S23 (Supporting Information) indicate that the overall morphology of nanorods and porous structure are maintained very well despite a long‐term (de)sodiation process. The well‐maintained pore structure, as displayed in Figure S24 (Supporting Information), demonstrates that the as‐designed substrate material can adapt the volume change and continuous redox reactions very well. The elemental distributions after cycling (Figure S25, Supporting Information) also suggest the stable existence and uniform distribution of S into the substrate material during electrochemical process. Note that the observed elemental F should be ascribed to decomposition of electrolyte to form an interface layer, suggesting the generation of side reactions.

The higher capacity, better cycling stability, and outperformed rate capabilities of the S/Nb_2_O_5_–CNR should be generally attributed to the joint action of unique micro/nanostructures of CNR and Na‐ion conductive orthorhombic Nb_2_O_5_ with excellent kinetical promotion. The Nb_2_O_5_‐functionalized nanoreactors with ultrafine porosity accommodates the active S and its volume change during cycling as well as functions as a chemical container for redox conversions of the active S. The synergy of physical confinement of CNR and chemical anchoring of polar Nb_2_O_5_ nanoparticles realizes both robust adsorption of NaPSs via Nb–S and Na–O bonds and their desorption, thereby inhibiting the shuttle effect and ensuring the following redox conversions. More importantly, orthorhombic Nb_2_O_5_ as a good Na‐ion conductor plays a crucial role in kinetically propelling electrochemical conversions from Na_2_S*_x_* to Na_2_S_2_/Na_2_S (Figure 5f).

The S loading content is a key parameter to determine the practical applications of S cathodes, so the loading content of S in the composite is further evaluated to 60% (denoted as S_60_/Nb_2_O_5_–CNR) to test its cycling performance. The as‐obtained S_60_/Nb_2_O_5_–CNR maintains a specific capacity of 520.5 mA h g^−1^ after 100 cycles at 0.1 A g^−1^ (Figure S26, Supporting Information). Compared with the S cathode with a loading content of 42%, the cycling capacity is evidently lower, suggesting the profound influence of S content in Na‐ion storage properties. In order to realize high‐loading and high‐performance S cathodes, further structural design and compositional regulation needs to be adopted in future research.

In consideration of superiorities of the as‐designed multifunctional nanoreactors in structures and components, we speculate that it may also allow much promise in universality for other battery systems with a multielectron redox electrochemistry. Thus, we select the emerging Na–Se batteries as an example to illustrate this point. The Nb_2_O_5_–CNR is composited with pristine Se to produce a Se/Nb_2_O_5_–CNR composite with a Se content of 56% (Figure S27a, Supporting Information), that is higher than most of current literature.^[^ ^52^, ^53^, ^54^, ^55^, ^56^, ^57^, ^58^ ^]^ The multielectron/phase‐involved stepwise redox mechanism can be revealed by the CV curves at a scan rate of 0.1 mV s^−1^ (Figure S27b, Supporting Information). The Se/Nb_2_O_5_–CNR composite cathode can exhibit a high initial specific capacity of 724 mA h g^−1^ at 0.1 A g^−1^ and maintains 403 mA h g^−1^ after 100 cycles (Figure S27c, Supporting Information). When evaluating its rate performance (Figure S27d, Supporting Information), reversible capacities of 609, 432, 415, 370, and 363 mA h g^−1^ are obtained at 0.1, 0.2, 0.5, 1.0, and 2.0 A g^−1^, respectively. Other than those, the Na–Se batteries harvest outstanding cyclability with a capacity retention of 273 mA h g^−1^ after 300 cycles at 0.5 A g^−1^ (Figure S27e, Supporting Information).

### 2.4 Electrochemical Evolution Mechanism of S Cathodes

The aforementioned discussion involves the structures and compositions, chemical affinity, kinetics properties, and electrochemical performance of the as‐designed materials. Here, in‐situ and ex‐situ analysis was also carried out in an attempt to getting deep insights into the sodium storage mechanism of the S cathode.

It was recognized by Liu et al.^[^ ^59^, ^60^ ^]^ and Yan et al.,^[^ ^48^, ^61^ ^]^ utilizing detailed characterization, comparison, and analysis, that the generation of polysulfides and parasitic nucleophilic side reactions would be involved at the early cycling process, but after building a stable electrolyte–electrode interface layer, the electrochemical evolution of the S cathode was dominant by quasi‐solid–solid reactions. Based on such a quasi‐solid redox mechanism, they further elucidated the distinguished definition of electrocatalysts for RT Na–S batteries with Li–S batteries.^[^ ^62^ ^]^ These advanced recognitions toward RT Na–S chemistries are very helpful for our following characterization and analysis.

The redox behaviors of the as‐assembled Na–S batteries is first investigated via CV tests (Figure S28, Supporting Information). A sharp peak around 2.05 V is usually attributed to electrochemical transformations from the pristine S to long‐chain polysulfides at the first cathodic scan process.^[^ ^47^, ^63^ ^]^ These polysulfides can be further converted into less soluble short‐chain polysulfides in the following reactions. Another evident small peak between 0.8 and 0.9 V is often regarded as the electrochemical signal corresponding to formation of Na_2_S.^[^ ^48^, ^64^ ^]^ There appear a series of irregular and irreversible impurity peaks between 1.0 and 1.6 V, mainly due to the side reactions from electrolyte solvent and polysulfides to form the solid–electrolyte interface (SEI) and cathode–electrolyte interface (CEI) layer. At the second cathodic scan, a highly repeatable peak centered at 1.6 V is believed to correspond to conversions from dissolved Na_2_S*_x_* (4 < *x* ≤ 8) to Na_2_S_4_.^[^ ^48^, ^65^ ^]^ A shifted peak between 1.2 and 1.4 V actually reflects the conversion from Na_2_S_4_ to short‐chain insoluble products compared with the first cycle, the shift of which may be caused by the unstable side reactions and electrochemical polarization. The broad shoulder peak at 1.0 V corresponds to the terminal reaction to generate Na_2_S. In all the scans, a substantial broad anodic peak positioning at about 1.9 V represents the oxidation process from Na_2_S_2_/Na_2_S to S, slight current attenuation of which suggests the same‐extent loss of S species during this process. These CV curves are also in good accordance with discharge–charge profiles. The difference in redox behaviors at the 1st–2nd cycle might be posed by decomposition of electrolyte and electrochemical activation of S species.^[^ ^38^, ^39^ ^]^

**Figure 6:**
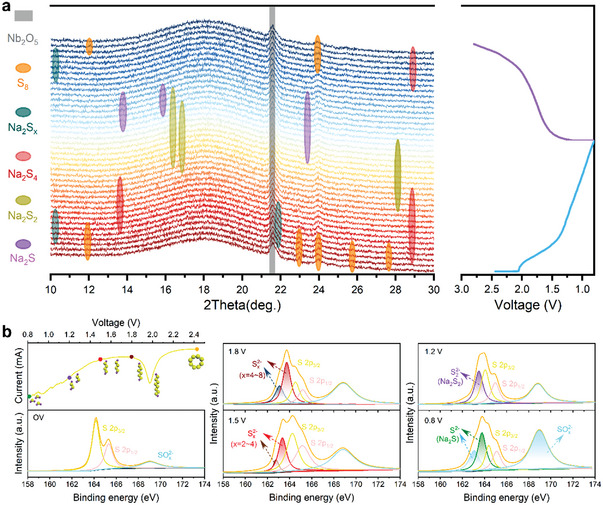
Electrochemical evolution mechanism. a) In‐situ XRD patterns accompanied with a discharge–charge profile at 0.1 A g^−1^. b) Cathodic CV curve and XPS spectra of S 2p at different discharge states.

To get further insight to electrochemical evolution of the S guest, in‐situ XRD was utilized to detect the phase changes of S during discharge–charge process at the first cycle. The S cathode with an elevated S content of 66% was alternatively employed for harvesting stronger phase‐change signals of cathode materials (**Figure** **6**a). The characteristic peaks were highly matched with that of S_8_ (PDF# 73–5065) at the OV state. As the main peaks of S vanished along with a lowered voltage (2.0 V), two obvious peaks located at ≈10.47° and 22° emerged, which is ascribed to formation of long‐chain polysulfides (Na_2_S*_x_*).^[^ ^66^ ^]^ Once the voltage was discharged to 1.5 V, two characteristic peaks attributed to Na_2_S_4_ (PDF# 71–0516), which positioned at 13.8° and 29°, respectively, could be clearly detected.^[^ ^60^, ^67^ ^]^ This indicates the transformations from long‐chain polysulfides to less soluble Na_2_S_4_. Afterward, three peaks at 16.7°, 17.1°, and 28.5° appeared when discharging to 1.2 V, which arose from the signals of solid‐phase Na_2_S_2_ (PDF# 81–1771). Following that, Na_2_S was continuously generated at the voltage range from 1.2 to 0.8 V, and finally, only three peaks of Na_2_S (PDF# 03–6920) could be observed, which located at 14.1°, 16.3°, and 23.4°, respectively. This clear phase change process fully confirms the electrochemical analysis from the abovementioned CV curves. As such, a multielectron/multiphase electrochemical evolution mechanism could be built in the designed RT Na–S batteries: S_8_ → Na_2_S*_x_* → Na_2_S_4_ → Na_2_S_2_ → Na_2_S. At the same time, during the charging process, featuring peaks from Na_2_S_4_, Na_2_S*_x_*, and S_8_ reappeared in turn, demonstrating the highly reversible redox reactions of the designed S cathode.

As is well‐known, XPS is sensitive to value state change of surface elements of materials. It was, therefore, employed to examine the S cathode at different discharging states (Figure 6b). At the OV state, XPS spectra of S 1s from the fabricated electrode exhibited three peaks corresponding to S 2p_1/2_, S 2p_3/2_, and SO*_x_* ^2−^, respectively. With discharging to 1.8 V, two new peaks emerged at binding energies of 163.10 and 163.75 eV, respectively. According to the previous reports,^[^ ^42^, ^68^ ^]^ it was caused by the formation of high‐order Na_2_S*_x_* (*x* = 4–8). When the voltage reached down to 1.5 V, the two peaks evidently shifted to 162.78 and 163.35 eV, respectively. This demonstrates that S species were further reduced to lower chemical values, which actually corresponded to conversions from high‐order polysulfides to low‐order sulfides (Na_2_S*_x_*, *x* = 2–4).^[^ ^15^ ^]^ As the sodiation proceeded, these complex polysulfides/sulfides were gradually reduced to Na_2_S_2_ and to the terminal Na_2_S.

These in‐situ and ex‐situ characterization results are in very good accordance with the previous reports about RT Na–S batteries and it is of greater importance that they sufficiently verify the multielectron/phase electrochemical evolution mechanism of S cathodes.

In summary, orthorhombic Nb_2_O_5_ incorporated into porous carbon nanoreactors (Nb_2_O_5_–CNR) was developed as a bidirectional redox regulator for RT Na–S batteries, which could accelerate reduction and oxidation conversions of S cathodes as well as promote solid‐phase transformations of low‐order sulfides via fast Na‐ion diffusion. Uniformly dispersed Nb_2_O_5_ onto carbon nanorods endowed the S cathode with abundant polar sites to prevent the irreversible loss of the active materials. The rod‐like porous carbon nanoreactors offered favorable physical confinement to the S guest due to their fine porous structure and adapted its dramatic volume change during (de)sodiation process. Benefitting by these multifunctional features of the designed material, the S/Nb_2_O_5_–CNR accomplished superior rate capability of 405 mA h g^−1^ at 2 A g^−1^ and excellent cycling stability (617 mA h g^−1^ after 600 cycles at 0.5 A g^−1^). It is worth pointing out that such a multifunctional host material is potentially extended to other similar guest materials (e.g., Se and Te). This work exploits a novel intrinsic metal oxide electrocatalyst and reveals its distinguished electrocatalytic mechanism with previous electrocatalysts (i.e., bidirectional catalysis). In addition to those, this work considerably sheds light on synergistic effect of micro/nanostructures and chemical compositions on reinforcing overall properties of S cathodes and we expect it can trigger the domino effect of developing metal oxide electrocatalysts for RT Na–S batteries.

## Conflict of Interest

The authors declare no conflict of interest.

## Supporting information

Supporting Information

## Data Availability

The data that support the findings of this study are available from the corresponding author upon reasonable request.
