# Supplementary material for: Orthorhombic Nb2O5 Decorated Carbon Nanoreactors Enable Bidirectionally Regulated Redox Behaviors in Room‐Temperature Na–S Batteries
Source: Adv Sci (Weinh). 2022 Dec 5;10(4):2206558. doi: 10.1002/advs.202206558 (PMC9896060; doi:10.1002/advs.202206558)
Supplement: Supplementary file 1 — Supporting Information [file ADVS-10-2206558-s001.pdf]

ADVANCED SCIENCE
Open Access

Supporting Information

for *Adv. Sci.*, DOI 10.1002/advs.202206558

Orthorhombic Nb_2O_5 Decorated Carbon Nanoreactors Enable Bidirectionally Regulated Redox Behaviors in Room-Temperature Na–S Batteries

Xiang Long Huang, Xiaofeng Zhang, Liujiang Zhou, Zaiping Guo*, Hua Kun Liu, Shi Xue Dou* and Zhiming Wang**

Supplementary Information

Orthorhombic Nb_2O_5 Decorated Carbon Nanoreactors Enable Bidirectionally Regulated Redox Behaviors in Room-Temperature Na-S Batteries

Xiang Long Huang,* Xiaofeng Zhang, Liujiang Zhou, Zaiping Guo,* Hua Kun Liu, Shi Xue Dou,* Zhiming Wang*

X.L. Huang, X. Zhang, Z. Wang
Institute of Fundamental and Frontier Sciences, University of Electronic Science and Technology of China, Chengdu 611731, China
E-mail: xlhuang_uestc@163.com; zhmwang@uestc.edu.cn

Prof. Z. Guo
School of Chemical Engineering & Advanced Materials, The University of Adelaide, Adelaide, SA 5005, Australia
E-mail: zaiping.guo@adelaide.edu.au

Prof. H.K. Liu and Prof. S.X. Dou
Institute for Superconducting and Electronic Materials, University of Wollongong, NSW 2500, Australia
Institute of Energy Materials Science, University of Shanghai for Science and Technology, Shanghai 200093, China
E-mail: shi@usst.edu.cn

Prof. L. Zhou
School of Physics, University of Electronic Science and Technology of China, Chengdu 611731, China

Materials and Methods

Synthesis of Nb_2O_5-CNR and CNR

600 mg of 2,5-dihydroxyterephtalic acid was immitted into a glass container with deionized water and dimethyl formamide (labelled as solution A). 1.008 g of zinc acetate dihydrate was immitted into the other container with deionized water and dimethyl formamide (labelled as solution B). Next, solution A was entirely poured into solution B and stirred for about half an hour to form a yellow suspension. Then, the yellow suspension was transferred into a Teflon-lined autoclave and hydro-thermally proceeded for 24 hours at 100 ˚C. After naturally cooling down to the ambient temperature, the yellow precipitate was collected via high-speed freezing centrifugation, purified with ethanol for three times at least, and dried overnight at 80 ˚C in a blast drying oven. The yellow solid powder was MOF-74, which was converted into a carbon nanoreactor (CNR) through carbonizing for 4.5 hours at 900 ˚C under the protective Argon gas flow. The yield of CNR was about 12%. 600 mg of the as-obtained CNR was dispersed into a transparent solution containing 450 mg of ammonium niobite (V) oxalate hydrate and 20 ml of deionized water to magnetically stir overnight, and dried for 12 hours at 50 ˚C in an oven to evaporate the water. Following that, the Nb-contained CNR powder was annealed for 3 hours at 800 ˚C with a heating speed of 10 ˚C/min. Finally, the resultant black powder was the target product Nb_2O_5-CNR.

Synthesis of S/Nb_2O_5-CNR and S/CNR

The identical mass of Nb_2O_5-CNR and sublimed sulfur was fully ground and then thermally treated for 12 hours at 155 ˚C in a closed container fulfilled with Argon gas to produce the S/Nb_2O_5-CNR composite. The control sample of S/CNR composite was prepared via the same method without any change.

Polysulfide adsorption experiment

A mixture of anhydrous sodium sulfide (Na_2S) and sublimed sulfur with a molar ratio of 1:5 was added into a mixed organic solution of 1, 3-dioxolane (DOL) and diethylene glycol dimethyl ether (DIGLYME) (v:v = 1: 1) and violently stirred for 12 hours under the protection of Argon gas to generate a Na_2S_6 solution with a concentration of 0.2 M. Then, the Na_2S_6 solution was diluted to a suitable concentration to carry out adsorption experiment. 40 mg of CNR and Nb_2O_5-CNR were added into the as-diluted Na_2S_6 solution, separately. The Na_2S_6 solution without any additives was used as a blank reference.

Materials characterization

The morphologies and nanostructures of all the samples were observed via field emission scanning electron microscope (ZEISS, GeminiSEM 300) and transition electron microscope (Tecnai G2 F20 S-TWIN). The elemental distributions were collected by energy dispersive spectrometry (EDS). The crystal structures and chemical states of all the samples were analyzed employing an X-ray diffractometer (XRD) with Cu K-alpha radiation and X-ray photoelectron spectra (XPS, Thermo Scientific ESCALAB 250Xi), respectively. Raman spectra of all the samples were obtained via InviaRefl (Renishaw, UK) with 532 nm laser light. The specific surface areas and pore distributions of all the samples were investigated by Brunauer-Emmett-Teller method (BSD PS2, China). The contents of compositions in the samples were estimated using

thermogravimetric analysis (TG 209, Germany) at corresponding gas atmospheres.

Electrochemical measurement

The prepared electrode materials, Ketjen black, and PVDF were mixed at a mass ratio of 8:1:1 in NMP solvent and grinded to a homogeneous slurry. The slurry was uniformly casted on a carbon-coated aluminum foil current collector, dried at 80°C for 12 hours in a vacuum oven to volatilize NMP. The diameter of disk electrodes was 12 mm. These disks made were assembled into 2032-type coin cells in an argon-filled glove box (oxygen/water contents are below 0.01 ppm). The diameter and thickness of the used sodium foils were 15.6 mm and 500 µm, respectively. The glass fiber disc (Whatman, GF1820-125) with a diameter of 19 mm served as separators. The electrolyte was 1M $NaClO_4$ dissolved into mixture solvent of EC and PC with 5% of FEC as an additive. The galvanostatic discharge-charge tests were conducted on a LAND instrument testing system under a room temperature. The voltage window employed was 0.8-2.8 V. CV curves were recorded on electrochemical workstation (Chenhua Instrument, CHI 660E) and EIS was also carried out on electrochemical workstation (Chenhua Instrument, CHI 660E) with a frequency range from 0.01 Hz to 100 kHz. The in-situ coin cell assembled with the S/Nb_2O_5-CNR composite was sealed by a Be foil, which was operated at a current density of 0.1 A g^{-1} and a voltage window of 2.8-0.8 V to acquire the in-situ XRD patterns.

First-principles calculation/simulation

The first-principle calculations were performed through the projector augmented wave (PAW) method and implemented in the Vienna ab initio Simulation Package (VASP). Generalized gradient approximation (GGA) parameterized by the form of Perdew-Burke-Ernzerhof (PBE) was adopted to describe the electron exchange correlation energy. The DFT-D3 method of Grimme was employed to characterize the van der Waals interaction. The energy cutoff for the plane waves was set to 400 eV. The Monkhorst-Pack method with 3×3×1 and gamma-centered k-points mesh were utilized for the Brillouin zone sampling of graphene and *T*-Nb_2O_5 (001) slab, respectively. Both the structural parameters and all the atoms were fully optimized until the Hellman Feynman forces were smaller than 0.05 eV/Å. A vacuum layer thickness of 15 Å suited to all the slab models. To describe the interaction between different substrates and polysulfides, the binding energy (E_b) can be described as $E_b = (E_{sub} + E_{ps}) - E_{(sub+ps)}$, where E_b and $E_{(sub+ps)}$ represents the energies of substrates without and with the adsorbed polysulfides, respectively, and E_{ps} is the energy of the isolated polysulfides. The charge density difference was calculated as $\Delta\rho = \rho_{sub+ps} - (\rho_{sub} + \rho_{ps})$, where ρ_{sub} and $\rho_{(sub+ps)}$ corresponds to the charge density of substrates without and with the adsorbed polysulfides, respectively, and ρ_{ps} is the charge density of the isolated polysulfides.

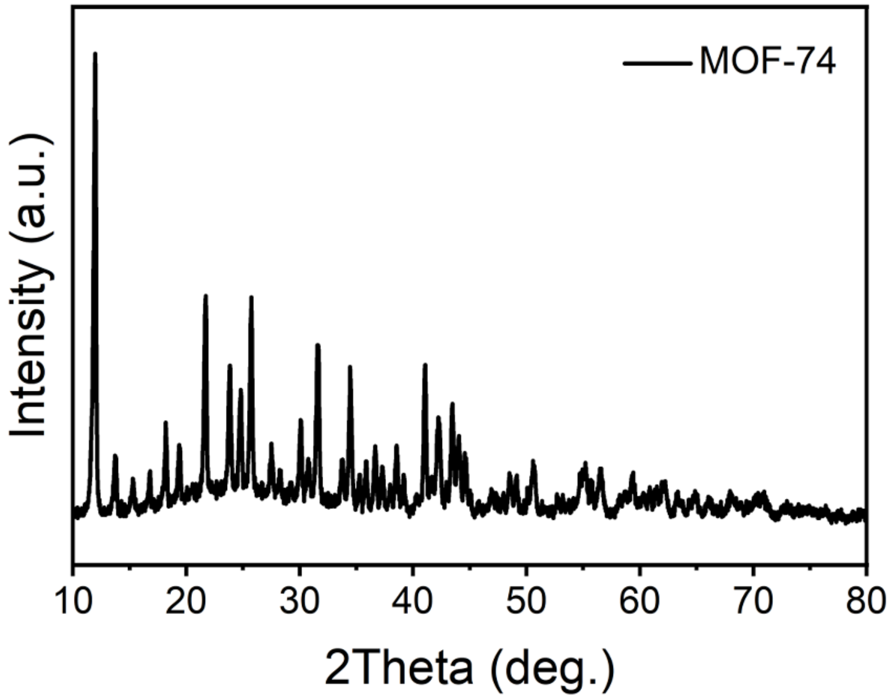

Figure S1. XRD pattern of MOF-74.

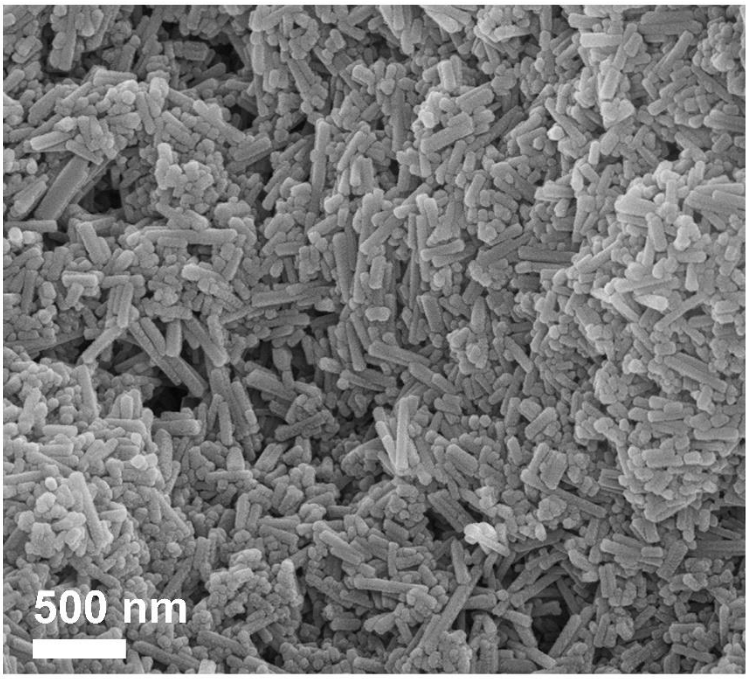

Figure S2. FESEM image of MOF-74.

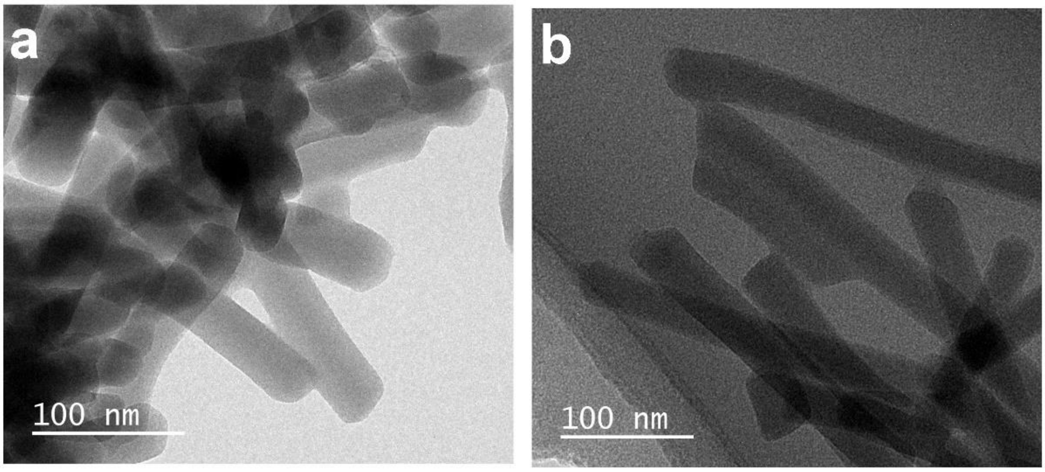

Figure S3. TEM images of MOF-74.

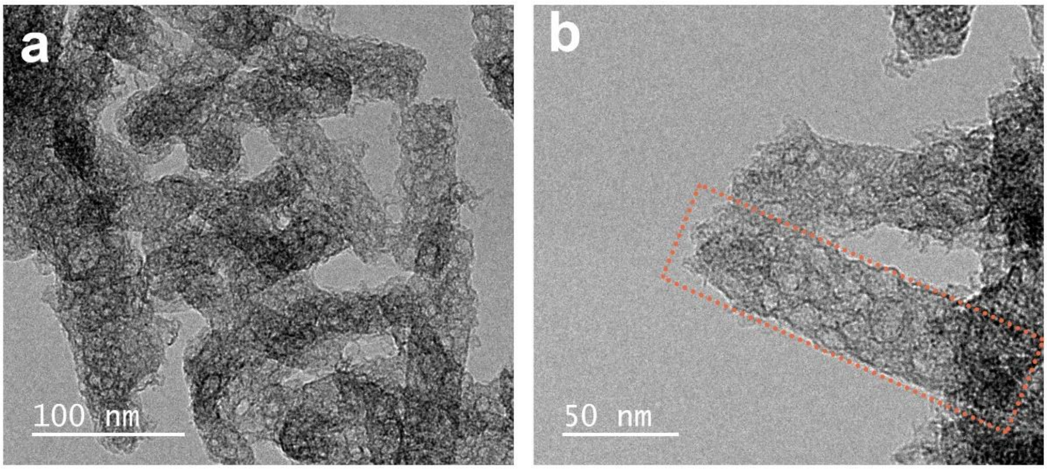

Figure S4. TEM images of CNR.

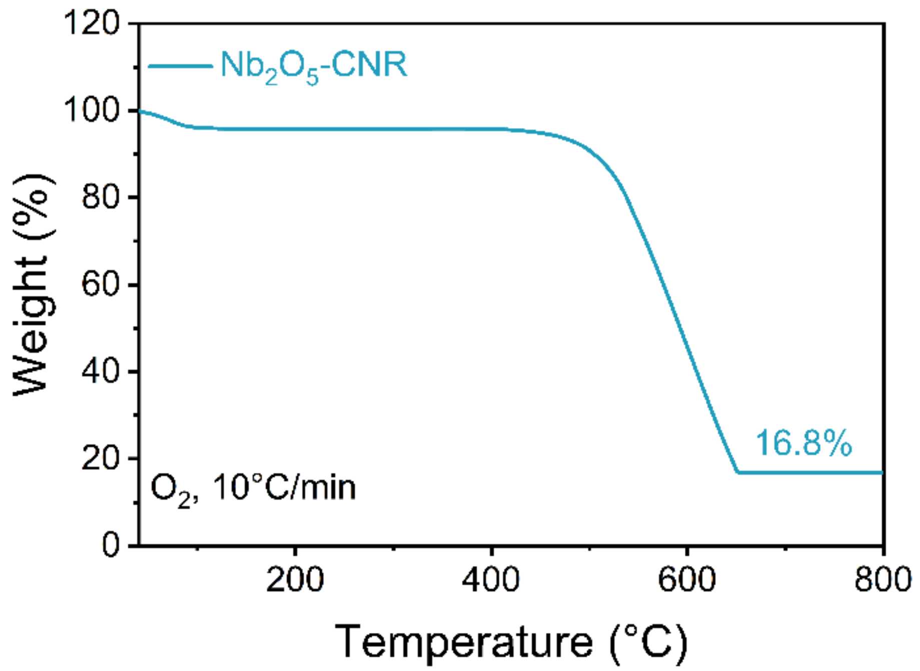

Figure S5. TGA curves of Nb_2O_5-CNR composite at O_2 atmosphere.

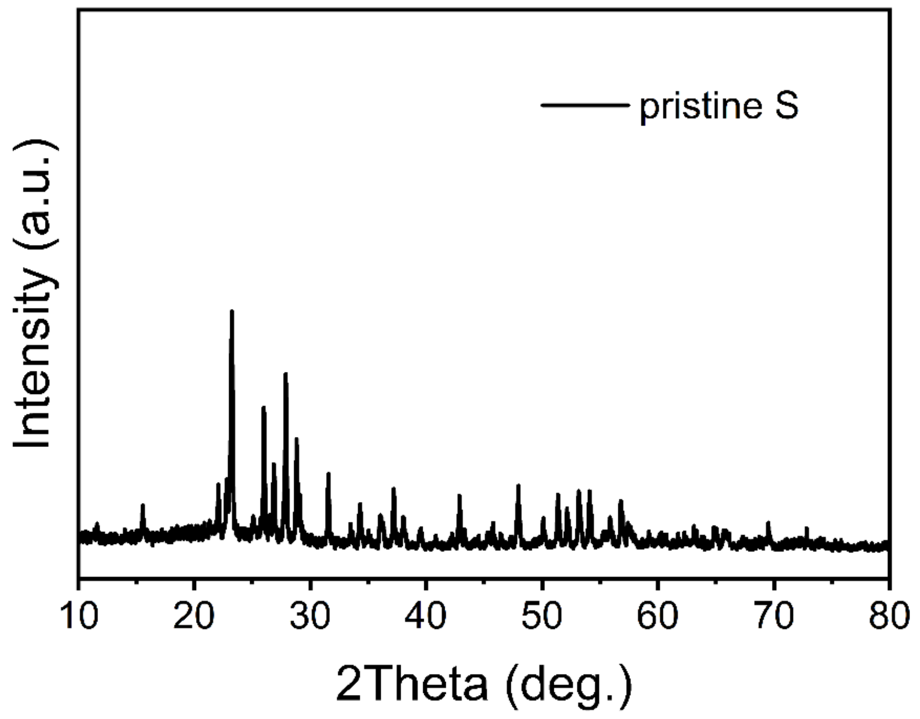

Figure S6. XRD pattern of sublimed sulfur.

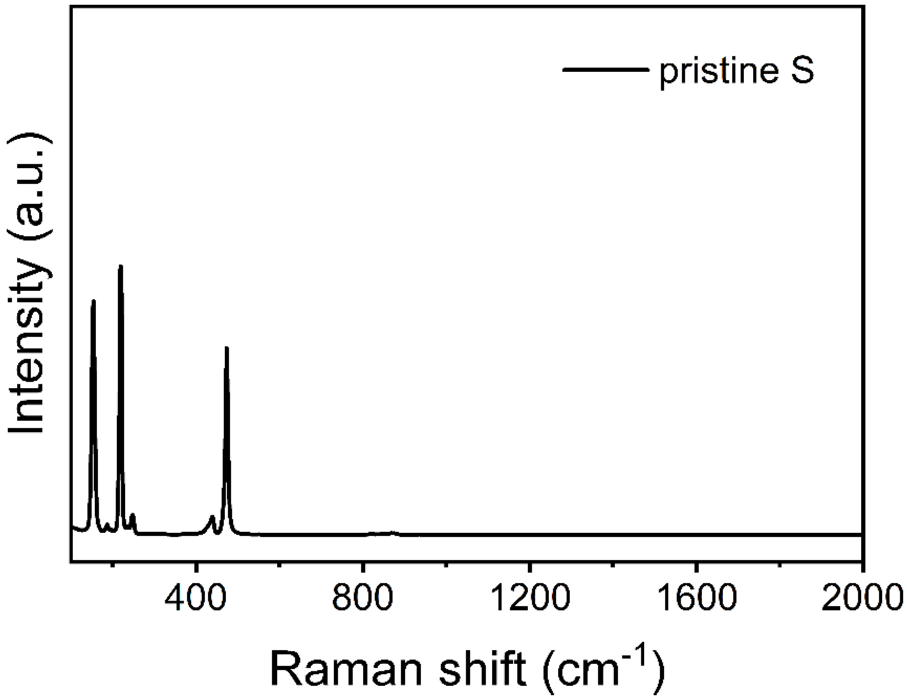

Figure S7. Raman spectra of sublimed sulfur.

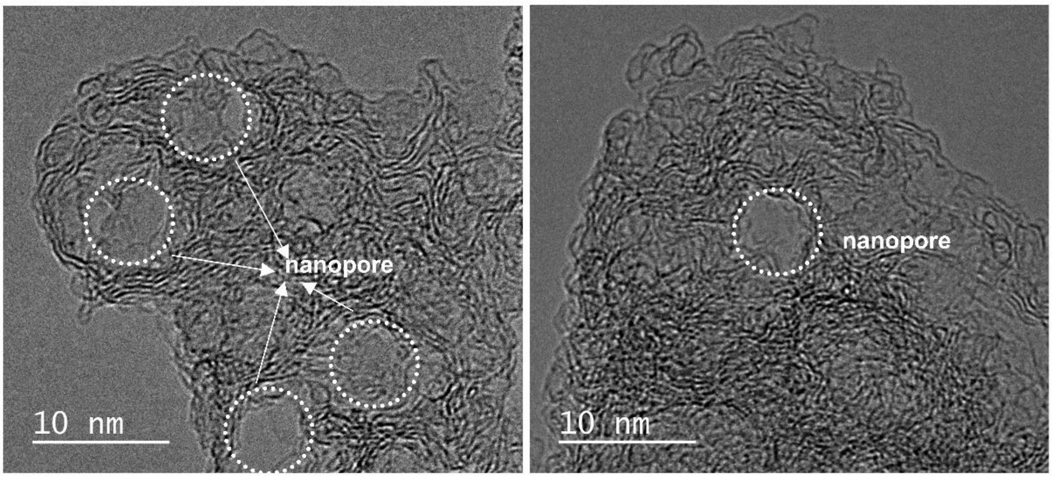

Figure S8. TEM images of the Nb_2O_5-CNR.

Table S1. A summary of porosity information of the samples.

Sample	Pore volume (cm^{-3} g^{-1})	Surface area (m^{-2} g^{-1})
CNR	1.778	928
Nb_2O_5-CNR	1.774	704
S/Nb_2O_5-CNR	0.702	75

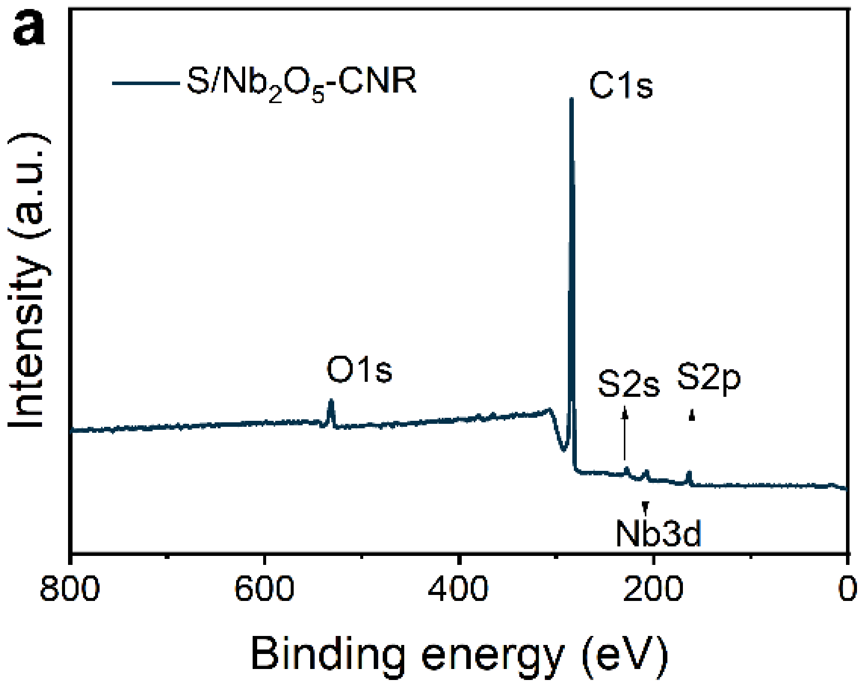

Figure S9. Wide-survey XPS spectra of S/Nb_2O_5-CNR.

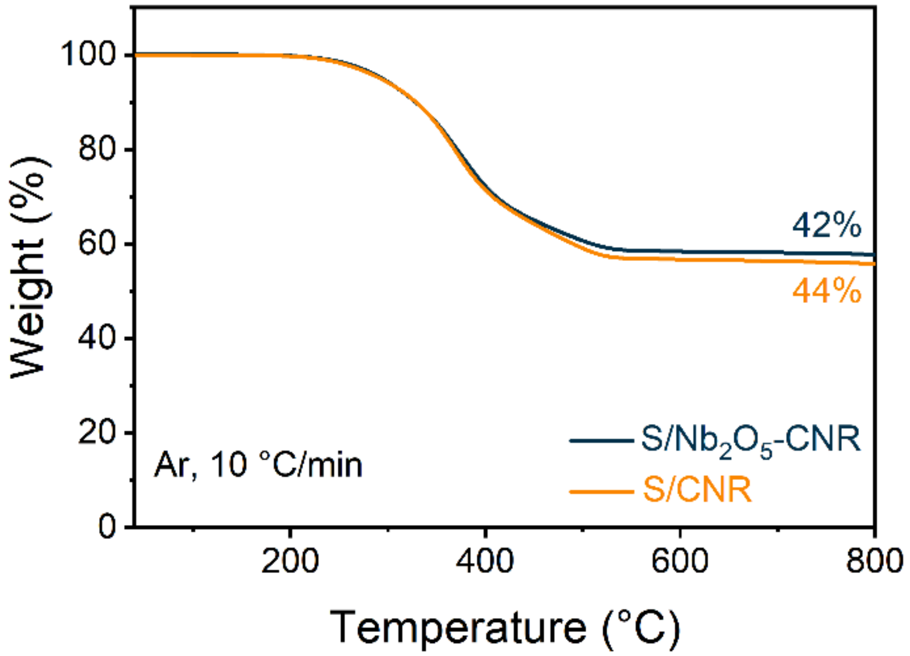

Figure S10. TGA curve of S/Nb_2O_5-CNR and S/CNR at Ar atmosphere.

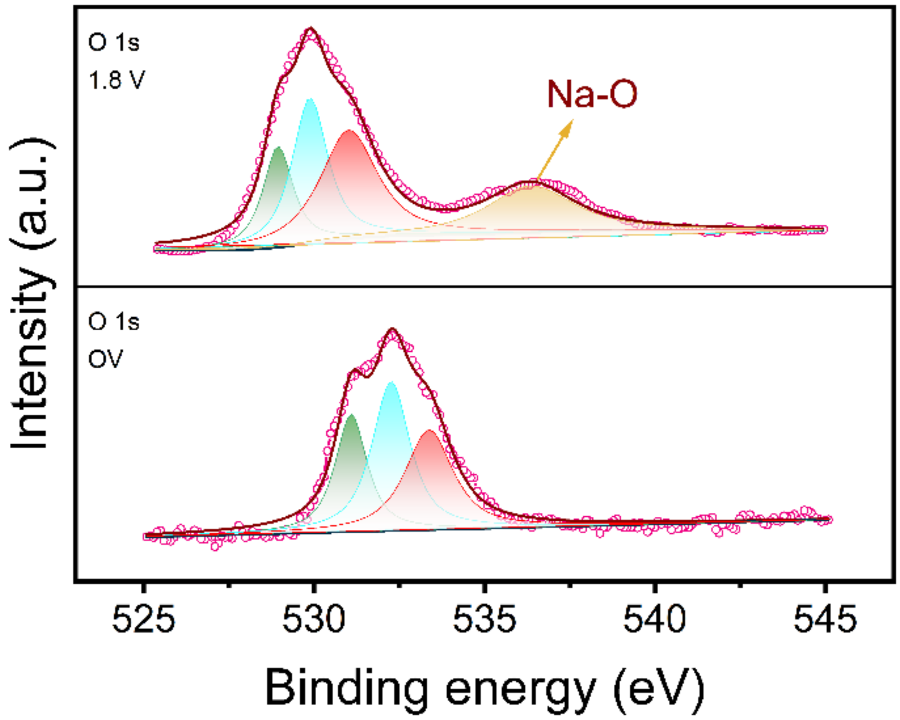

Figure S11. XPS spectra of O 1s before and after discharging to 1.8 V.

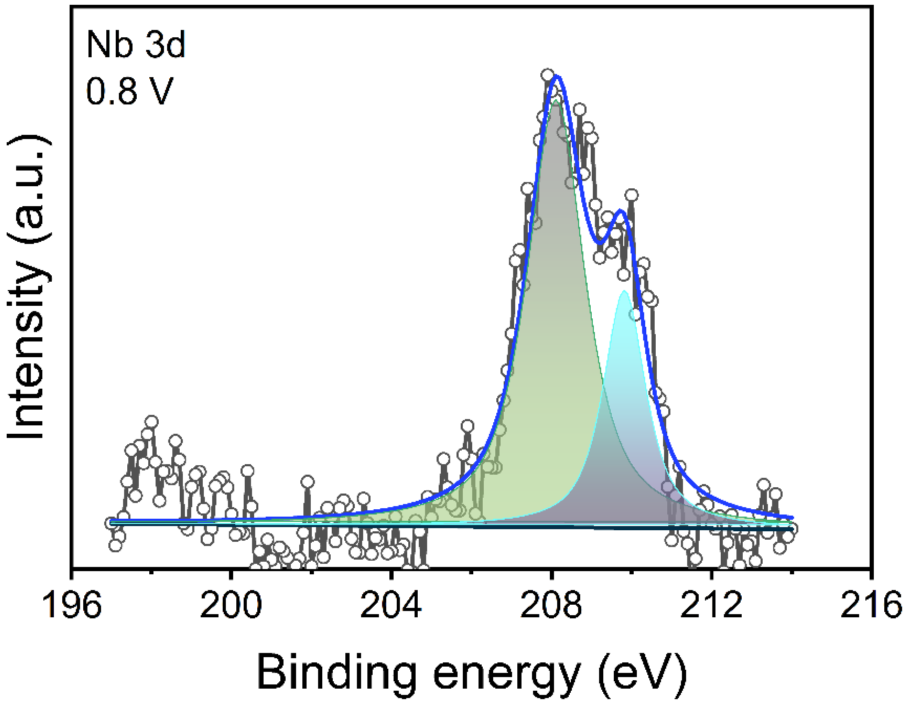

Figure S12. XPS spectra of O 1s after discharging to 0.8 V.

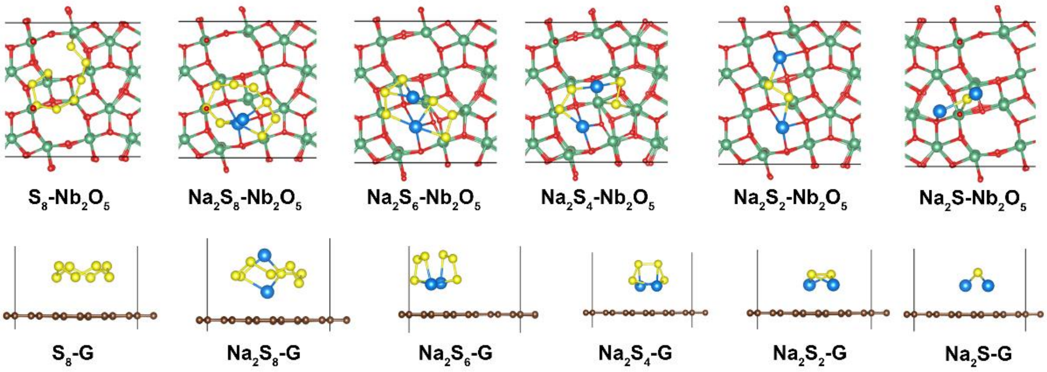

Figure S13. Conformations of different Na_2S_x species on the Nb_2O_5 and graphene.

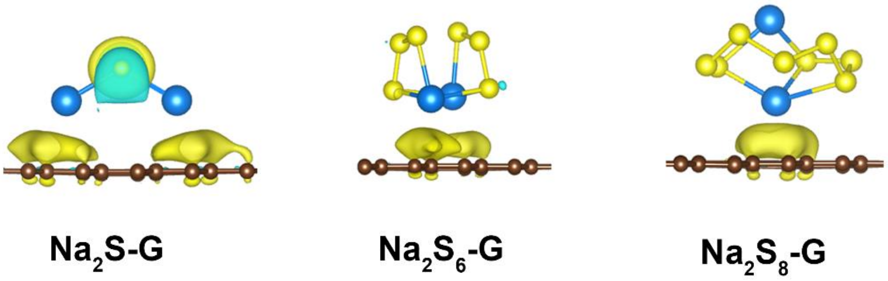

Figure S14. Charge density distribution for Na_2S, Na_2S_6, and Na_2S_8 molecules adsorbed on the graphene.

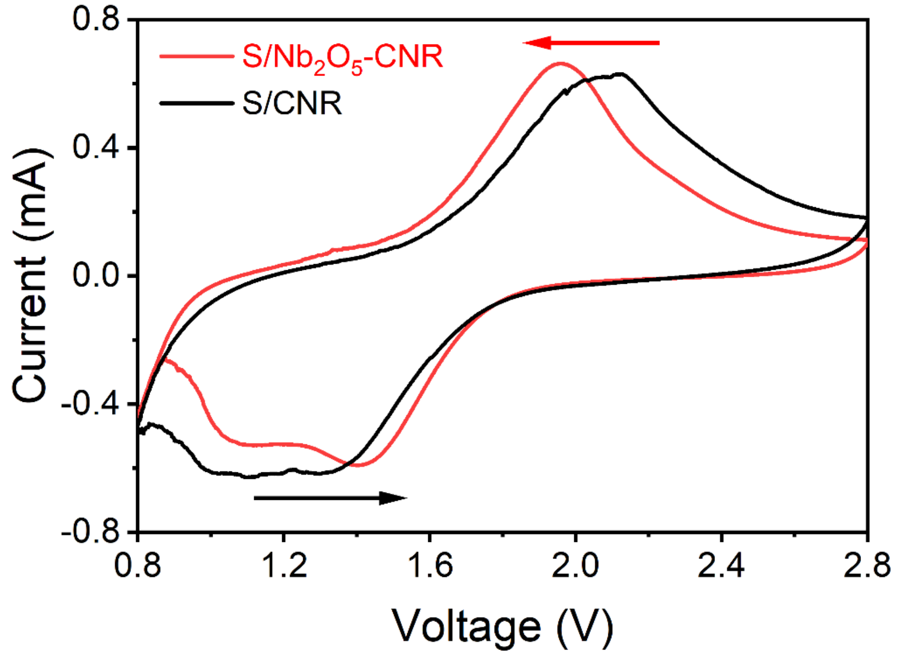

Figure S15. CV curves of the S/Nb_2O_5-CNR and S/CNR at the same scan rate.

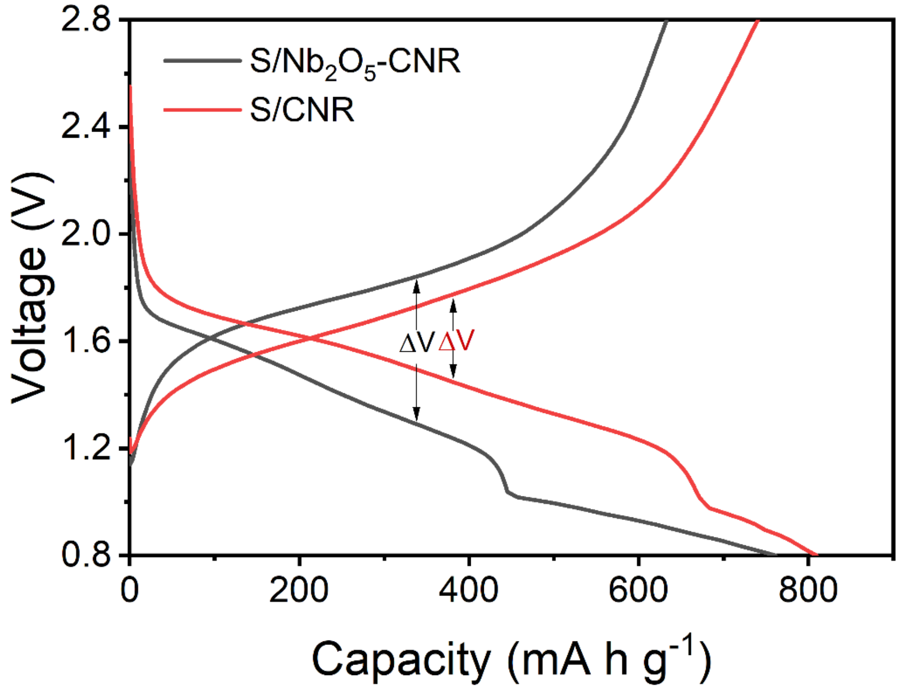

Figure S16. Discharge-charge profiles of the S/Nb_2O_5-CNR and S/CNR at the same rate.

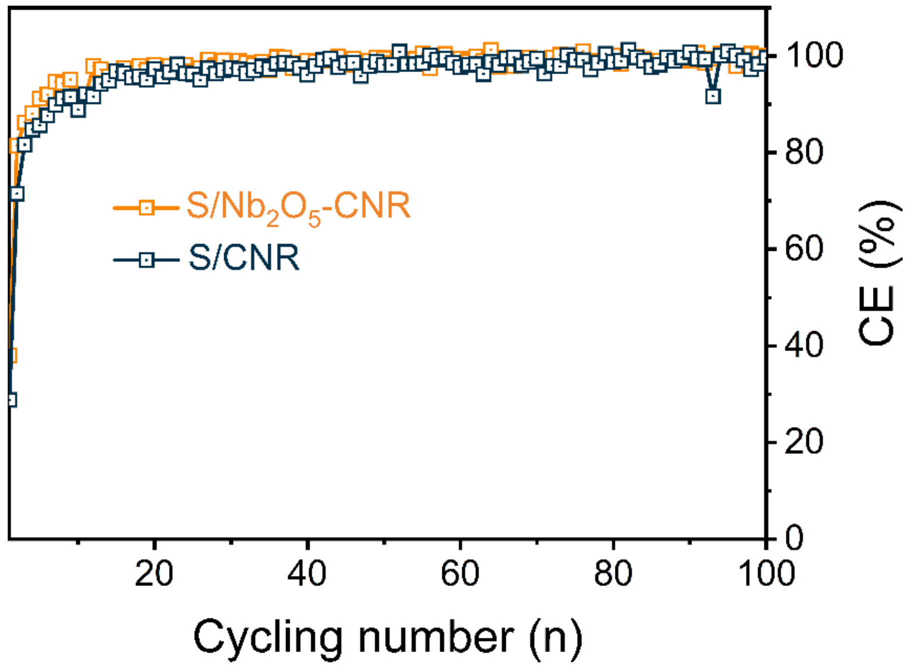

Figure S15. CE of cycling process of S/CNR and S/ Nb_2O_5-CNR at 0.1 A g^{-1}.

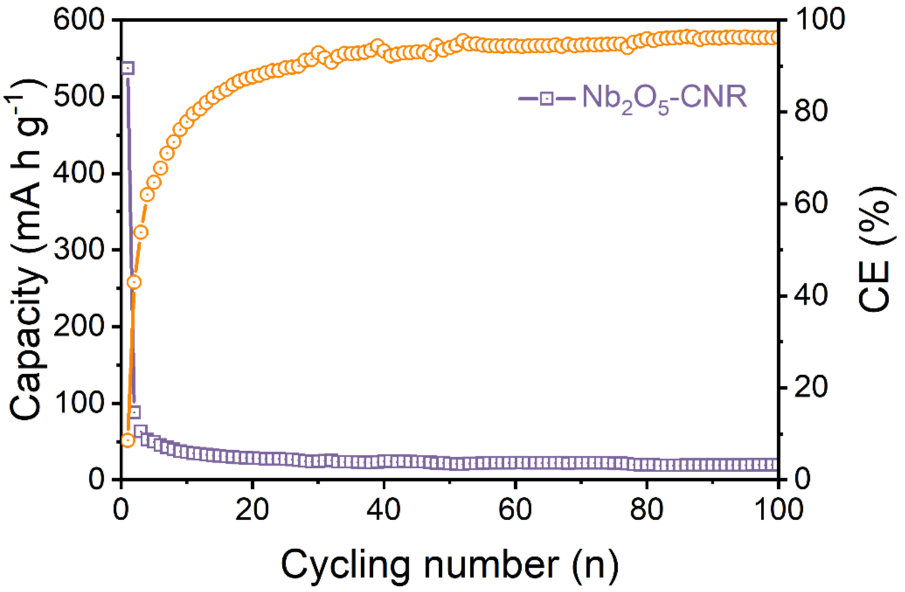

Figure S16. Cycling performance of Nb_2O_5-CNR at 0.1 A g^{-1}.

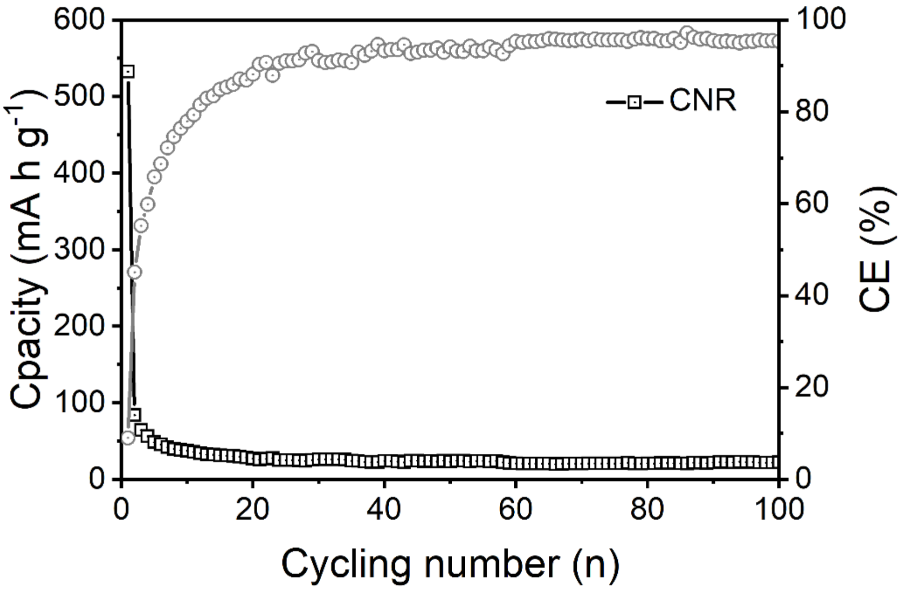

Figure S17. Cycling performance of the CNR at 0.1 A g^{-1}.

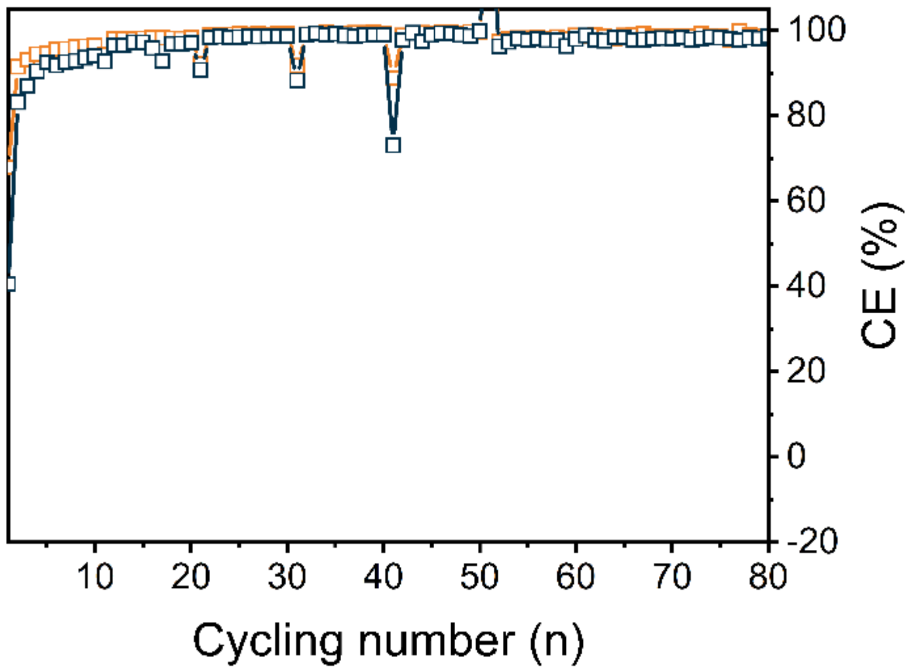

Figure S18. CE along with rate test process at various rates.

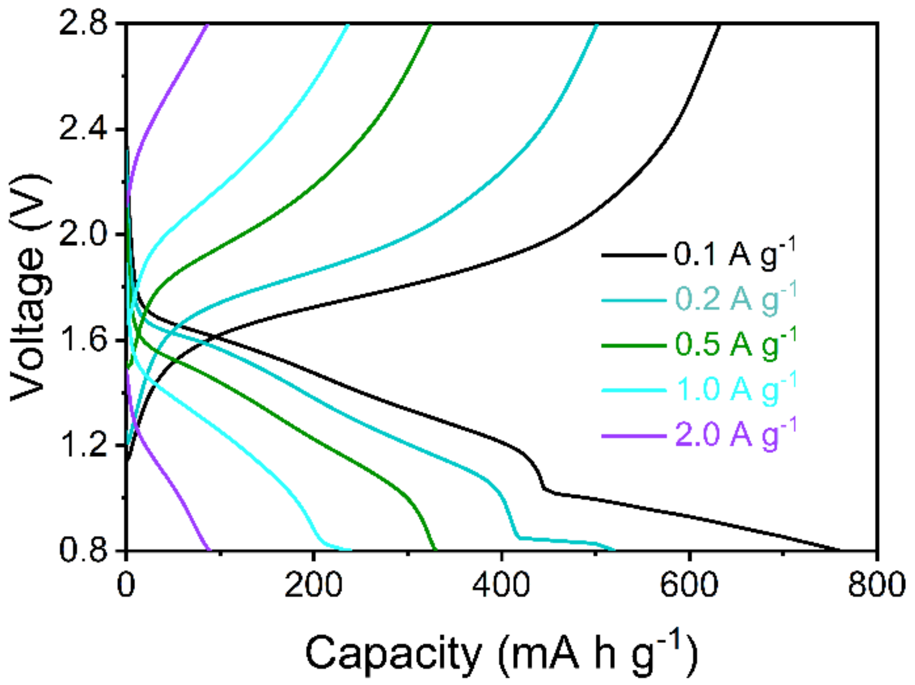

Figure S19. Discharge-charge profiles of the S/CNR at various rates.

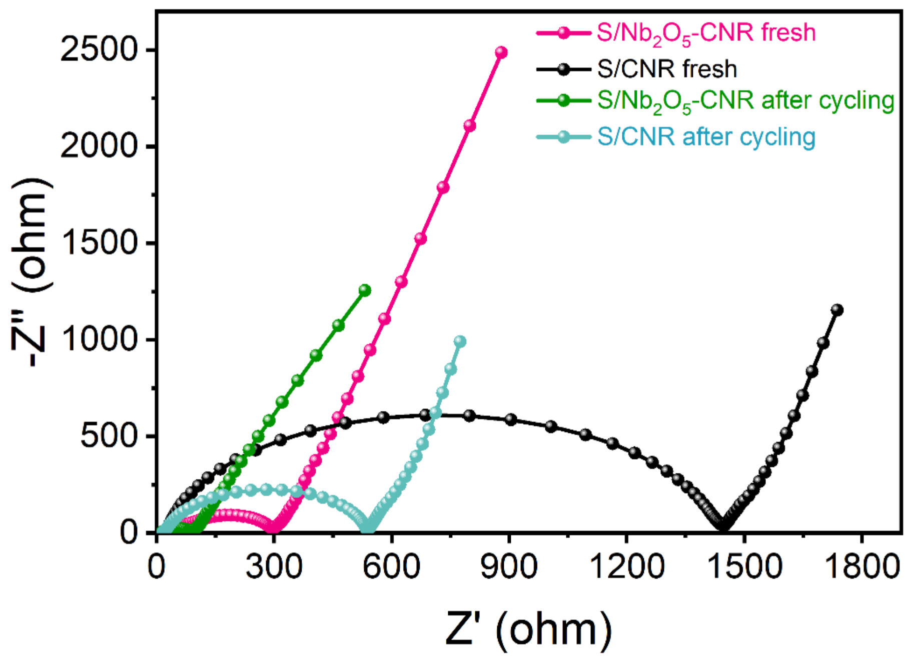

Figure S20. EIS spectra of S/Nb_2O_5-CNR and S-CNR before and after cycling.

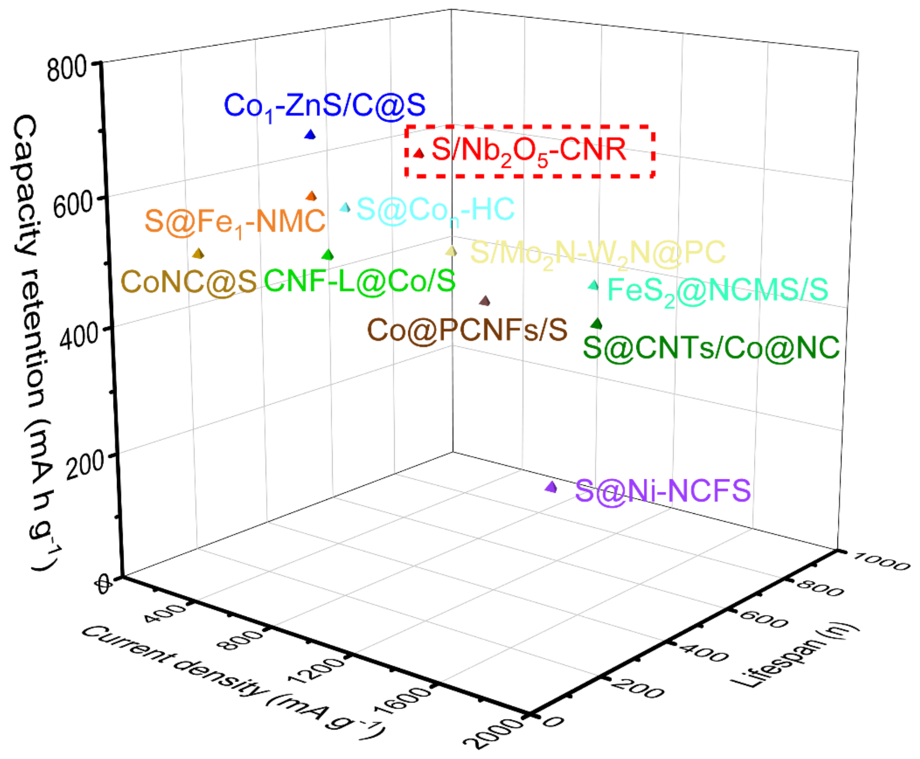

Figure S21. Prolonged cycling performance comparison of catalyst-decorated S cathodes.

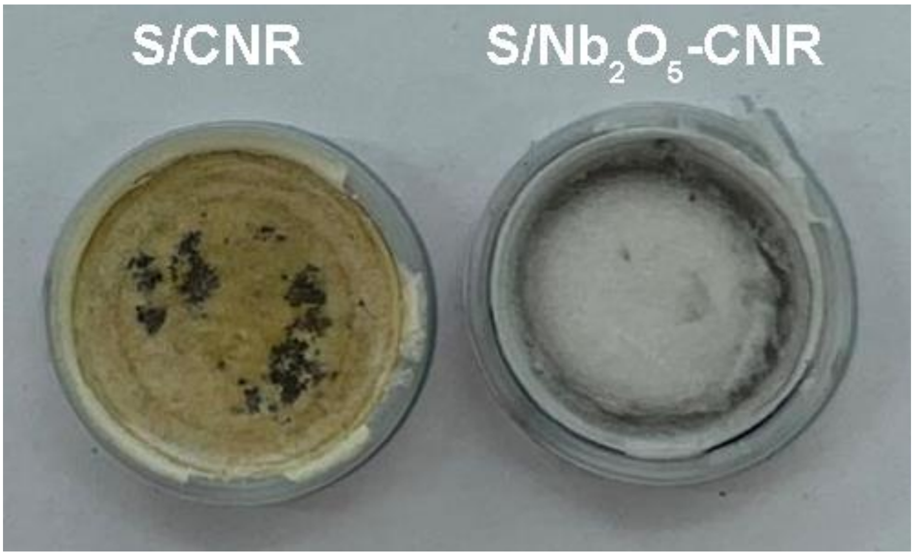

Figure S22. Separators of Na-S cells with the S/CNR and S/Nb_2O_5-CNR after cycling.

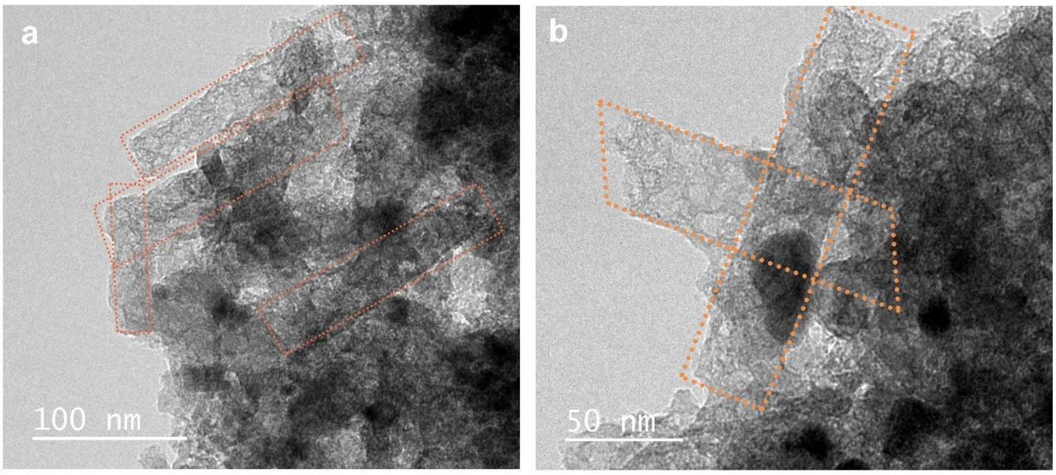

Figure S23. TEM images of the S/Nb_2O_5-CNR composite after cycling.

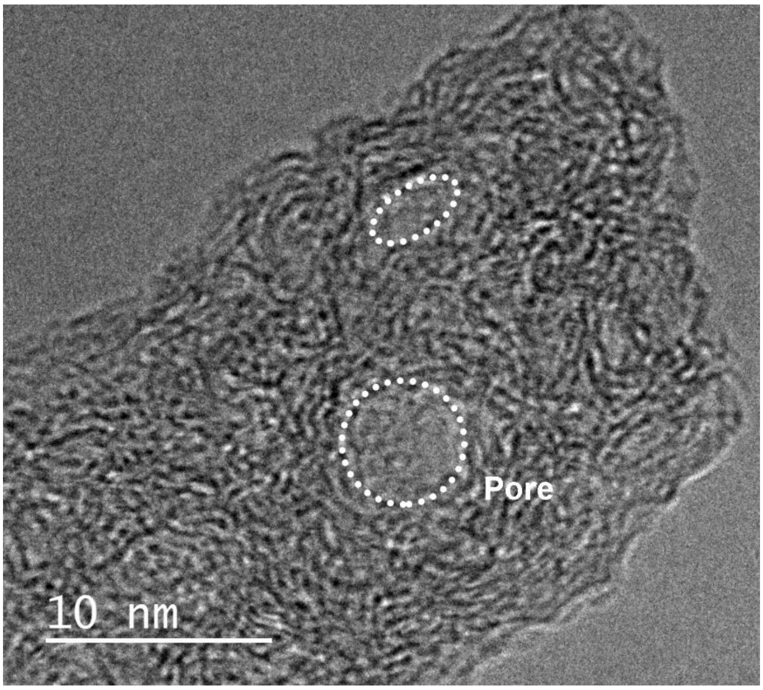

Figure S24. HRTEM image of the S/Nb_2O_5-CNR composite after cycling.

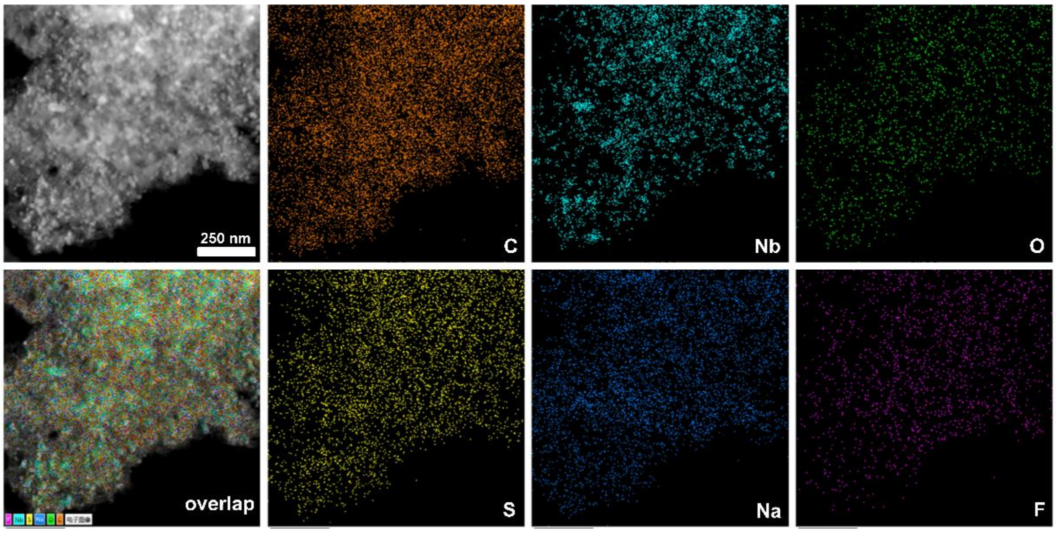

Figure S25. STEM image and corresponding EDS mappings of the S/Nb_2O_5-CNR composite after cycling.

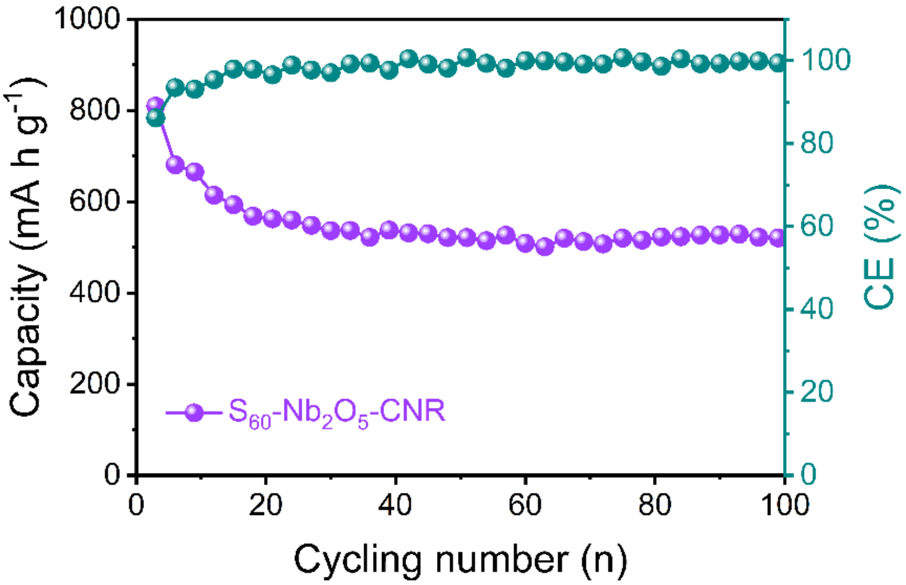

Figure S26. Cycling performance of the S_{60}/Nb_2O_5-CNR.

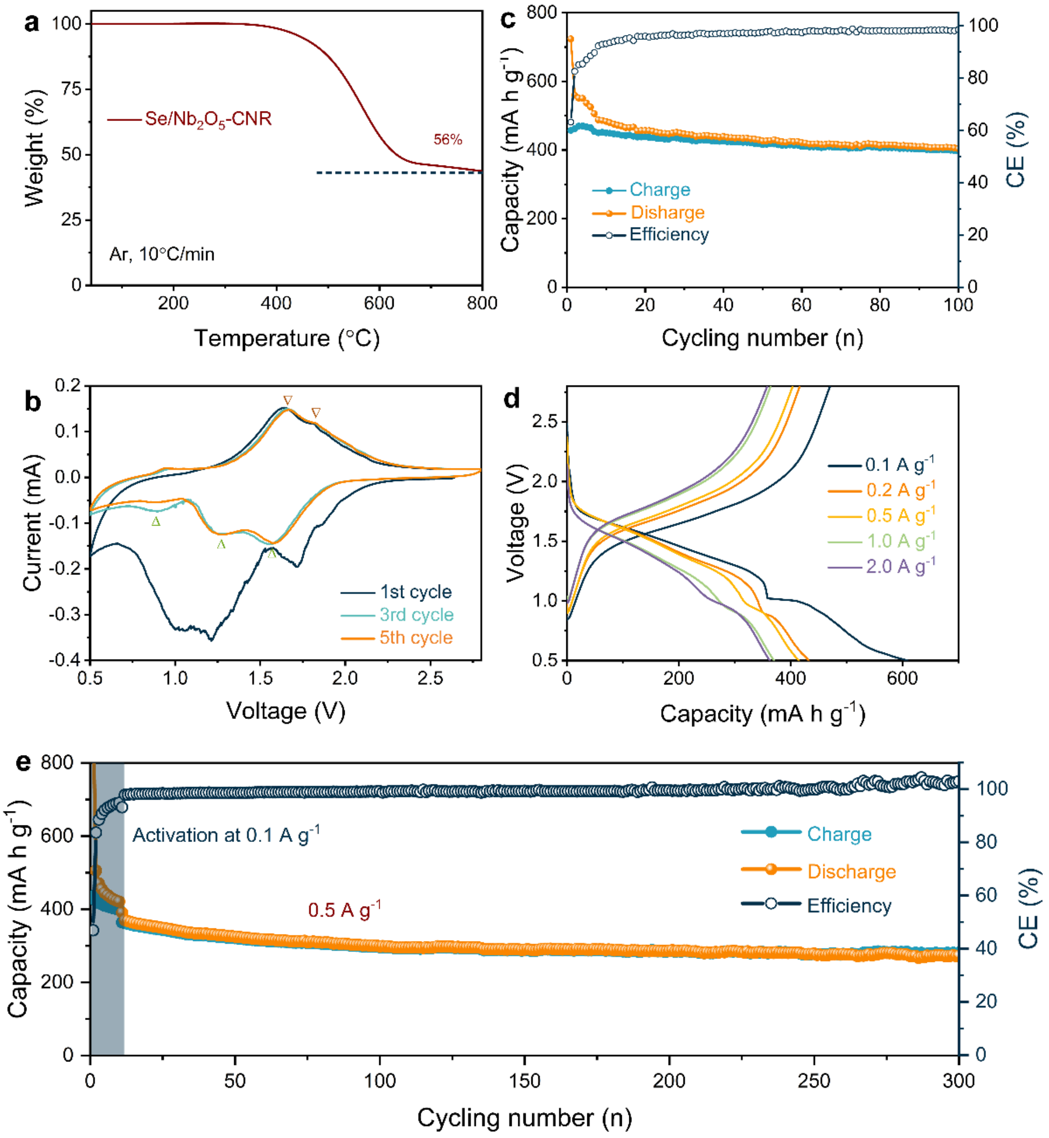

Figure S27. Electrochemical performance of the Se/Nb_2O_5-CNR.

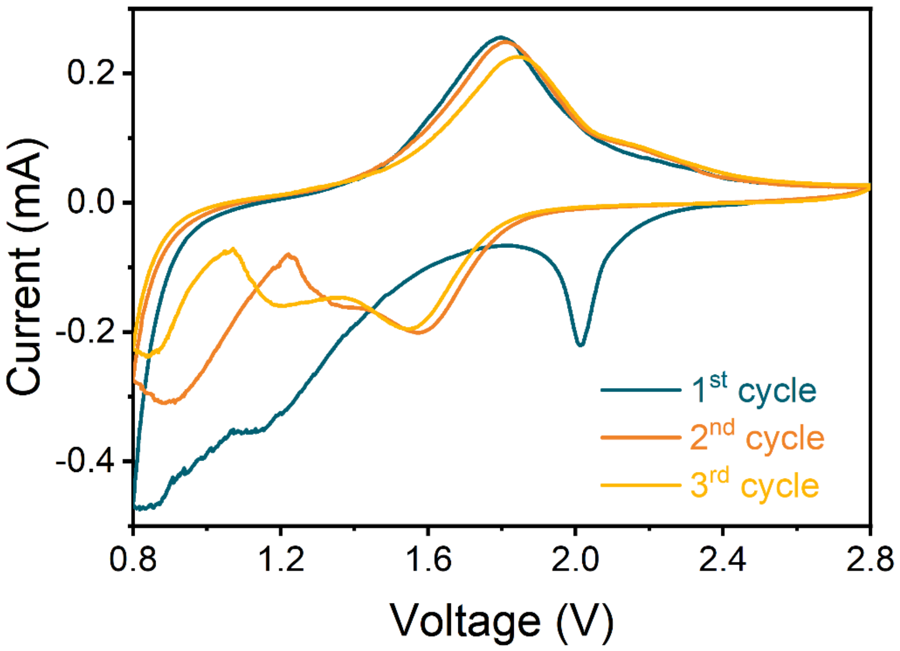

Figure S28. CV curves of the S/Nb_2O_5-CNR at a scan rate of 0.1 mV s^{-1}.

References for the Computational method:

1. Kresse, G. & Joubert, D. From ultrasoft pseudopotentials to the projector augmented-wave method. Phys. Rev. B 59, 1758-1775 (1999).

2. Kresse, G. & Furthmuller, J. Efficient iterative schemes for ab initio total-energy calculations using a plane-wave basis set. Phys. Rev. B 54, 11169-11186 (1996).

3. Kresse, G. & Furthmuller, J. Efficiency of ab-initio total energy calculations for metals and semiconductors using a plane-wave basis set. Comp. Mater. Sci 6, 15-50 (1996).

4. Perdew, J. P., Burke, K. & Ernzerhof, M. Generalized gradient approximation made simple. Phys. Rev. Lett. 77, 3865-3868 (1996).

5. Grimme, S., Antony, J., Ehrlich, S. & Krieg, H. A consistent and accurate ab initio parametrization of density functional dispersion correction (DFT-D) for the 94 elements H-Pu. J. Chem. Phys. 132, 154104 (2010).

6. Monkhorst, H. J., Pack, J. D. Special points for Brillouin-zone integrations. Phys. Rev. B 13, 5188-5192 (1976).
